# Overexpression of *SHORT VEGETATIVE PHASE-LIKE (SVL)* in *Populus* delays onset and reduces abundance of flowering in field-grown trees

**DOI:** 10.1038/s41438-021-00600-4

**Published:** 2021-08-01

**Authors:** Greg S. Goralogia, Glenn T. Howe, Amy M. Brunner, Emily Helliwell, Michael F. Nagle, Cathleen Ma, Haiwei Lu, Amanda L. Goddard, Anna C. Magnuson, Amy L. Klocko, Steven H. Strauss

**Affiliations:** 1 Department of Forest Ecosystems and Society, Oregon State University, Corvallis, OR USA; 2 Department of Forest Resources and Environmental Conservation, Virginia Tech, Blacksburg, VA USA; 3 Department of Biology, University of Colorado Colorado Springs, Colorado Springs, CO USA

**Keywords:** Molecular engineering in plants, Field trials

## Abstract

The spread of transgenes and exotic germplasm from planted crops into wild or feral species is a difficult problem for public and regulatory acceptance of genetically engineered plants, particularly for wind-pollinated trees such as poplar. We report that overexpression of a poplar homolog of the floral repressor *SHORT VEGETATIVE PHASE-LIKE (SVL)*, a homolog of the Arabidopsis MADS-box repressor *SHORT VEGETATIVE PHASE (SVP)*, delayed the onset of flowering several years in three genotypes of field-grown transgenic poplars. Higher expression of *SVL* correlated with a delay in flowering onset and lower floral abundance, and did not cause morphologically obvious or statistically significant effects on leaf characteristics, tree form, or stem volume. Overexpression effects on reproductive and vegetative phenology in spring was modest and genotype-specific. Our results suggest that use of *SVL* and related floral repressors can be useful tools to enable a high level of containment for vegetatively propagated short-rotation woody energy or pulp crops.

## Introduction

Mitigation or prevention of gene flow from transgenic or gene-edited plants, especially in cases where hybridization to wild relatives is possible, may be needed to deploy these plant cultivars in the field^1^. This is especially important for forest tree species, which may be less intensively managed compared to other crops, have wide seed and pollen dispersal, have critical ecosystem functions, and may be cultivated in close proximity to wild species that could produce viable hybrids^2,3^. Plantations of forest trees often consist of exotic species, which may become invasive depending on management practices and environment^4,5^. For many angiosperm tree species, vegetative propagation is used to produce elite cultivars for outplanting^6^. In these cases, suppression of flowering and seed production could reduce or eliminate gene flow between plantation forests and their wild counterparts without sacrificing, and even potentially enhancing, wood productivity^7,8^.

Several strategies for reproductive containment have been demonstrated or proposed for forest trees. These strategies largely focus on eliminating functional flowers by modifying floral development, eliminating viable pollen or seed due to meiotic disturbance (e.g., selected polyploidy or aneuploidy), or using the controlled expression of cytotoxic factors to prevent pollen or ovules from maturing^7,9,10^. Additionally, the inserted transgenes could be excised during pollen or ovule development to prevent transgene inheritance^11,12^. Strong reproductive containment could potentially be achieved by preventing the initiation of inflorescence primordia, initiation of floral primordia, or development of floral organs^8,13,14^. In the former case, trees would not produce any inflorescence or floral structures. In the latter cases, plants would develop inflorescences, but no fertile flowers would form. When floral onset is prevented or disrupted very early in the development of inflorescence primordia, vegetative biomass may be increased as little energy or nutrients would be wasted on reproductive tissues^7^.

Flowering in *Populus* occurs after a long period of juvenility, typically several years to decades depending on environment and genotype^15^. Male catkins produce an abundance of pollen that is dispersed by wind, and fertilized female catkins produce seeds with cottony exteriors to aid in dispersal by wind or along waterways. Inflorescence development is specified in mid-spring under long-day conditions along newly extending shoots^16^. Catkin floral development occurs under protected inflorescence bud scales, but is temporarily suspended during winter dormancy. In early spring, flowers specified the previous year emerge prior to vegetative bud burst and are primarily located on the exterior of the tree crown^15^.

The molecular underpinnings of flowering onset and floral development have been extensively studied in *Arabidopsis thaliana* (Arabidopsis) and other model species (reviewed in Pin and Nilsson)^17^. While genes that regulate flowering in perennials are not as well characterized, many homologs of key Arabidopsis flowering genes have similar roles in species of *Populus*, *Malus*, and *Prunus*^16,18,19^. Based on the Arabidopsis model, *FLOWERING LOCUS T* (*FT*) encodes a key morphogenic peptide required for the transition to flowering and is produced within the phloem companion cells of leaf vascular tissue once developmental, photoperiodic, and temperature requirements for flowering are met (reviewed in Andrés and Coupland)^20^. *FT* function is well conserved in trees, such that overexpression leads to rapid flowering—often years before normal flowering would begin^21–23^. However, it is unknown whether the elevated expression of an *FT* suppressor, such as *SVL*, could prevent or delay the onset of flowering in a tree.

Several repressors of *FT* expression belong to a family of MADS-box-containing transcription factors that include *FLOWERING LOCUS C* (*FLC*), *SHORT VEGETATIVE PHASE* (*SVP*), *FLOWERING LOCUS M* (*FLM*), and the *MADS AFFECTING FLOWERING* (*MAF2-5*) proteins in Arabidopsis^24^. Overexpression of these floral MADS-box repressors typically results in a delay of flowering in annual plants, particularly under short-day conditions when photoperiodic activators are not expressed (reviewed in Castelán-Muñoz et al.)^25^. Conversely to the effect found in annuals, a recent study of homologs similar to *SVP* in apple revealed late vegetative bud burst in spring when *MdSVP* was overexpressed, but normal flower development and time to first flowering^19^. More recent studies in apple with *SVP* and the similar *DORMANCY ASSOCIATED MADS (DAM)* genes showed altered dormancy but not a delay in time to first flowering^26,27^. Another recent study in poplar demonstrated that *SHORT VEGETATIVE PHASE-LIKE (SVL)* is a repressor of bud burst following experimentally induced endodormancy^28^. The same study also showed that poplar *FT1* is a direct target for repression by SVL. For poplar, it is unknown if *SVP*-like genes have a function in flowering time suppression. Based on a multiple-year field study of three poplar clones overexpressing *Populus trichocarpa SVL*, we report that overexpression is associated with a multiple-year delay in the onset of flowering, and a reduction in floral abundance once flowering begins. As phenology determines the length of the growing season, one concern based on the delayed bud flush of *35:SVL* poplars is reduced productivity when used as tools for genetic containment^28^; in fact ectopic expression of the repressor *CENTRORADIALIS1 (CEN1*) delayed bud flush and greatly reduced growth in the field^29^. Our studies, however, showed that *SVL* overexpression caused a strong delay in first onset and intensity of reproduction, but had a non-significant effect on phenology, leaf morphology, or vegetative productivity.

## Results

### Identification and overexpression of *SHORT VEGETATIVE PHASE-LIKE (SVL)*

**Fig. 1:**
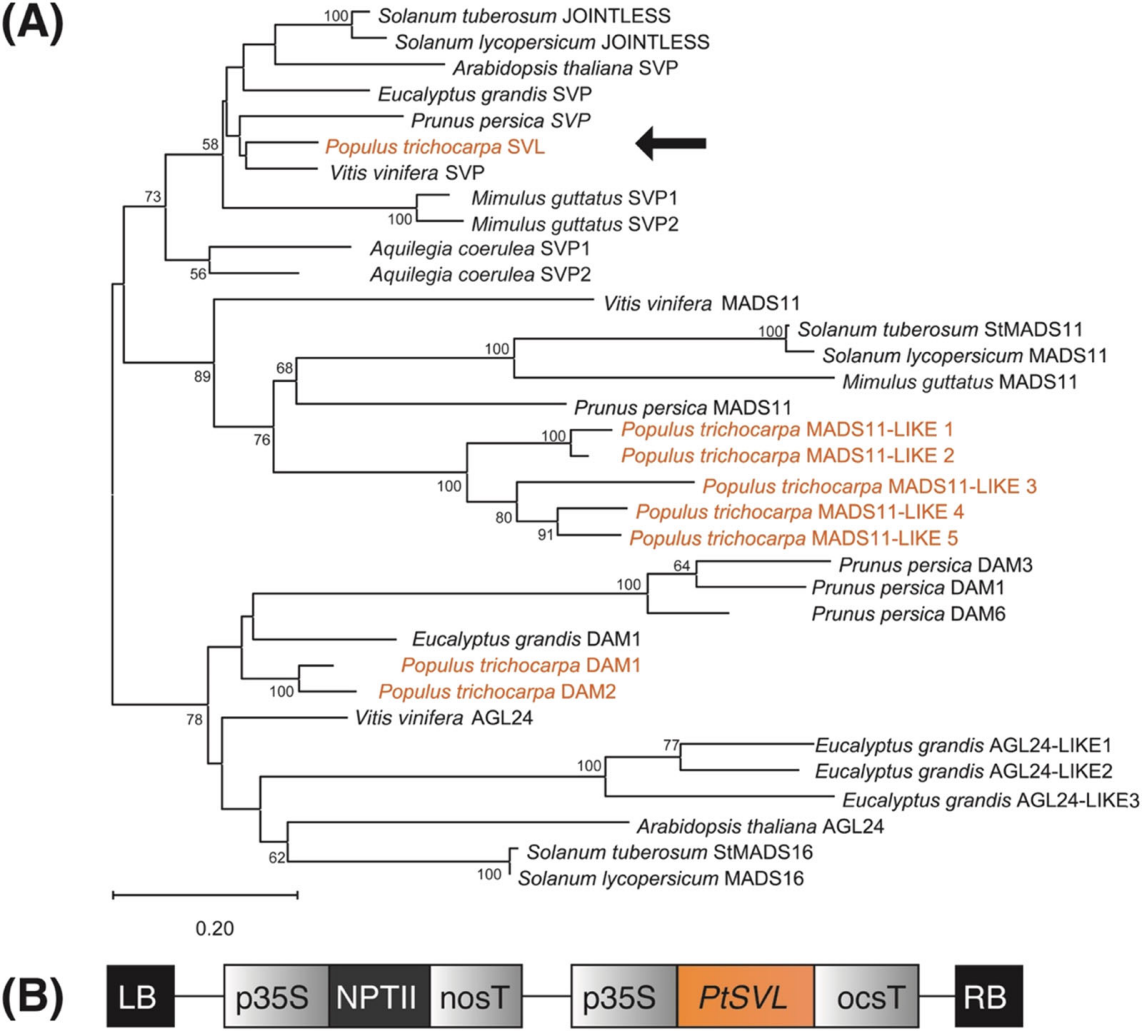
Identification of candidate *Populus trichocarpa* SVL homologs and overexpression construct design. **A** Phylogeny of eudicot proteins in the StMADS11 superclade^26^. Representative eudicot members of the StMADS11 superclade were obtained by BLAST P searches of the following Phytozome v13 (https://phytozome-next.jgi.doe.gov/) proteomes (protein ID prefix): *Populus trichocarpa* v4.1 (Potri, shown in orange), *Aquilegia coerulea* v3.1 (Aqcoe), *Solanum lycopersicum* ITAG3.2 (Solyc), *Solanum tuberosum* v4.03 (PGSC), *Mimulus guttatus* NONTOL v4.0 (MgNOTOL), *Eucalyptus grandis* v2.0 (Eucgr), *Vitis vinifera* v2.1 (VIT), *Prunus persica* v2.1 (Prupe), *Arabidopsis thaliana* Araport11 (AT). Bootstrap values equal or greater than 50% are shown at nodes. Full length protein sequences were aligned with MUSCLE^45^. A maximum likelihood phylogenetic analysis was performed on the sequence alignment using the JTT + G model, 75% deletion of alignment gaps/missing data and 100 bootstraps for branch support testing with the program MEGA X^46^. Arrow indicates *P. trichocarpa* protein chosen for overexpression in this study. **B** Architecture of the *SVL* overexpression construct. LB T-DNA left border, p35S Cauliflower mosaic virus 35S promoter, nosT nopaline synthase terminator, ocsT octopine synthase terminator, RB T-DNA right border

Because of its role in the suppression of flowering initiation genes, we sought to identify the closest *Populus trichocarpa* homolog to the Arabidopsis *SVP* gene. *SVP* and *AGL24* are the only Arabidopsis members of the *StMADS11* superclade, whereas the *Populus trichocarpa* genome contains nine members (Fig. 1A)^30^. However, only one poplar gene groups in the *SVP* subclade. The coding sequence of the SVP ortholog, referred to as SVL, was cloned from *P. trichocarpa cv. “*Nisqually-1”, and then placed downstream of a *35S* promoter with duplicated enhancer elements and upstream of an octopine synthase terminator (Fig. 1B)^31^. Expression values for *SVL* and the closest related MADS-box genes in *P. trichocarpa* in several tissue types are shown in Suppl. Fig. 1^32^.

The resulting *SVL* overexpression construct was transformed into three poplar genotypes. These include a female hybrid clone, 717-1B4 *P. tremula x alba*, a second female clone, 6k10 *P. alba*, and a male hybrid clone 353-38 *P. tremula x tremuloides*. Hereafter, 717-1B4 is abbreviated as “717”, and 353-38 as “353”. A total of 45 independent transgenic events were obtained, clonally propagated, and then planted in a field trial near Corvallis, Oregon in the summer of 2011. Four ramets were planted for each event to test the effects of various sterility and reproductive containment genes on flowering and growth^8,13^.

### *35S:SVL* transgene expression and field observations over eight years of growth

**Fig. 2:**
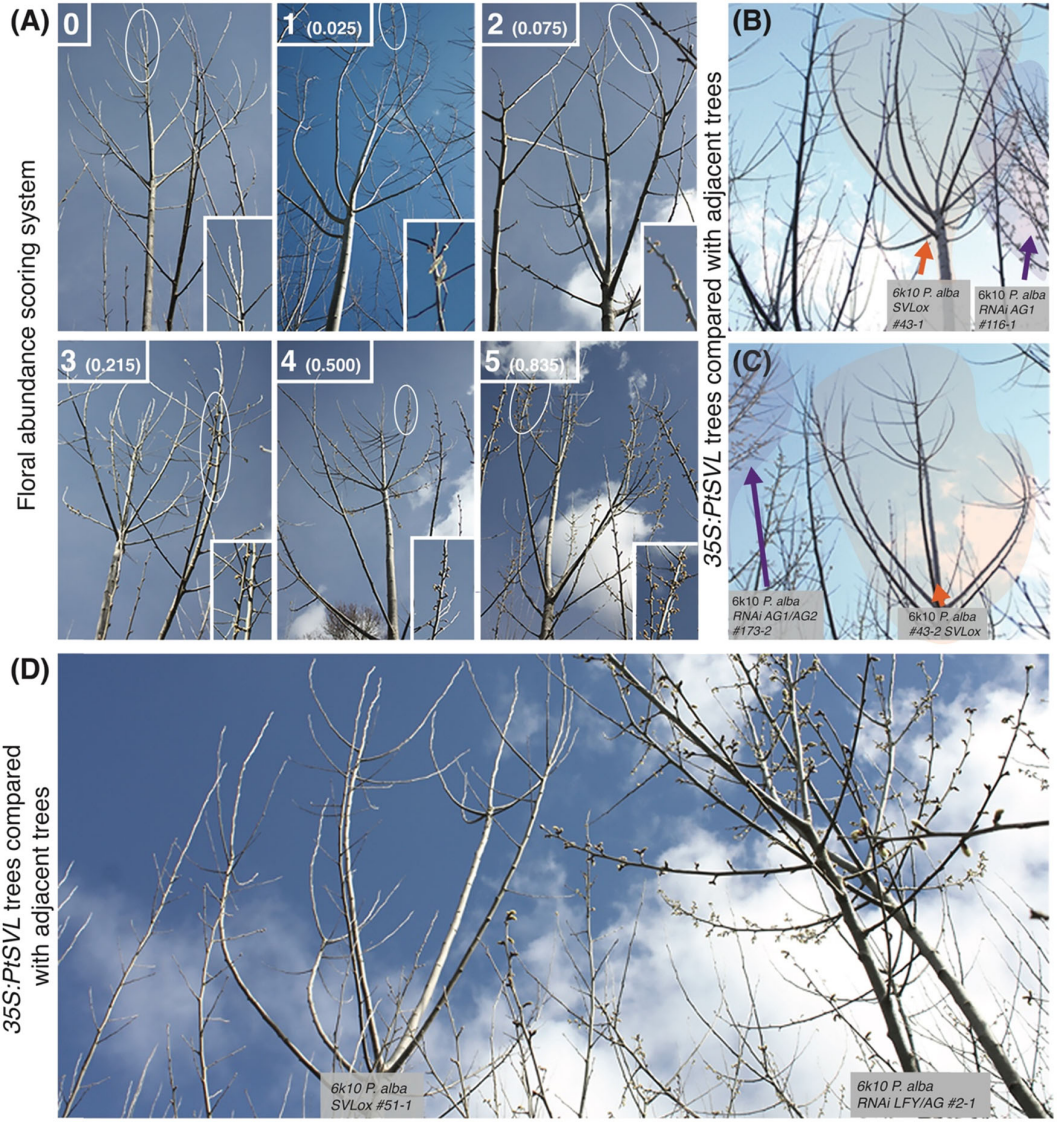
Representative images of field grown *SVL* overexpressing transgenic poplars. **A** Examples of field grown poplars illustrating the floral scoring system used to determine flowering presence and abundance prior to bud break in spring. Trees were scored from 0 to 1, then converted for quantitative analysis as 0 to 0.835 (see “Methods” section) based on the increasing coverage of catkins within the crowns of established trees. **B**–**D** Representative examples of *SVL* overexpressors with delayed flowering adjacent to flowering individuals of the same age produced with constructs that did not appear to affect onset or abundance of flowering. Flowering neighboring transgenic trees from unrelated constructs in **B** and **C** are indicated by purple arrows. Non-flowering *35S:SVL* transgenics are indicated by an orange arrow. Images were taken in the spring of 2017, 6 years after field planting, with a Canon Rebel XSI digital camera

Survival of trees at the field site was 95% from 2011 to 2019 over all clones^8^. We began measuring floral abundance in 2014, when most of the trees in the plantation began to flower. In 2016–2019, when flowering was common and intense in many trees, we noted many examples where flowering was absent in the *35S*:*SVL* trees, but abundant in adjacent trees (Fig. 2B–D). Whereas the adjacent trees were also transgenic and contained constructs designed to induce infertility, these neighboring trees were determined to have floral onset similar to non-transgenic controls based on phenotypic assessments^8^. Although we did not notice any obvious phenotypic differences in the vegetative appearance of *35S*:*SVL* trees vs. comparators, we did note a tendency for reduced secondary branching in the upper crowns of the *35S*:*SVL* trees (Fig. 2B–D).

**Fig. 3:**
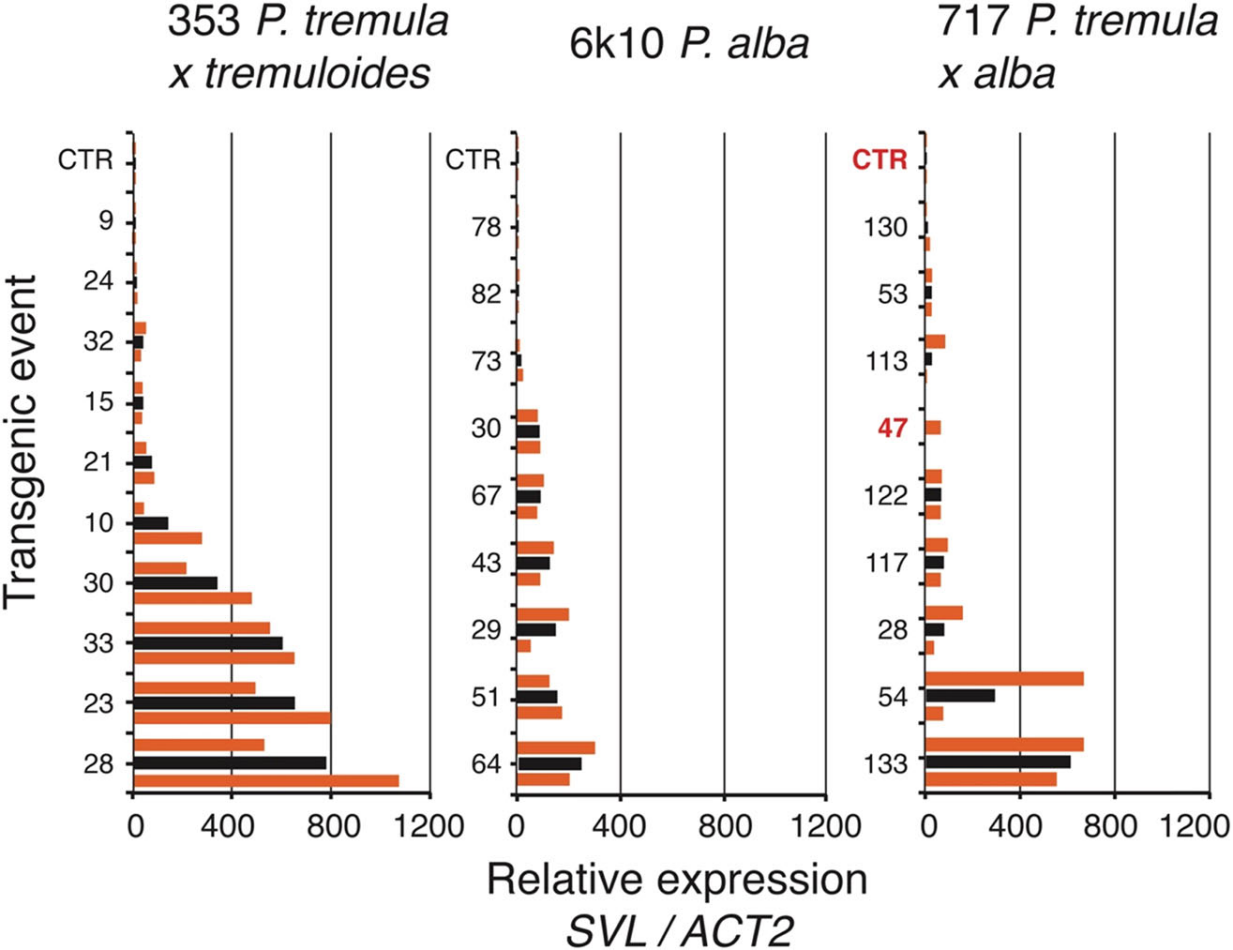
Relative expression of *SVL* in overexpressing poplar transgenic lines. RNA was extracted from leaves of three poplar clones (353, 6k10, 717) in the spring of 2017. Numbers indicate individually transformed lines (events) and CTR indicates the untransformed control line. For each line, the orange bars show the relative expression for each of two genetically identical ramets (mean of three technical replicates per ramet). The black bars show the mean of the two ramets for each line. An amplicon of the constitutively expressed *ACTIN2 (ACT2)* gene (Potri. 004G153400) was used to determine relative expression level of *SVL*. Two events (highlighted in red) were excluded from subsequent statistical analysis

We assessed *SVL* gene expression in control and transgenic trees in the spring of 2018 and found that our overexpression construct led to increased expression in all three clones tested. The events with the highest expression were found in the 353 clonal background (Fig. 3). In the 717 background, we found a few events with high expression and many events with low expression (Fig. 3). In most instances, *SVL* expression was similar in both ramets of the same event, but in a few instances (e.g., 353:28, 717:54), *SVL* expression differed substantially between ramets (Fig. 3). After accounting for mixed effects involving clones, constructs, and blocks, we found that 70% of the variance in gene expression occurred among events and 30% occurred among ramets within events (i.e., including variation among ramets within and among blocks).

### *SVL* expression is correlated with delayed floral onset and decreased floral abundance

**Fig. 4:**
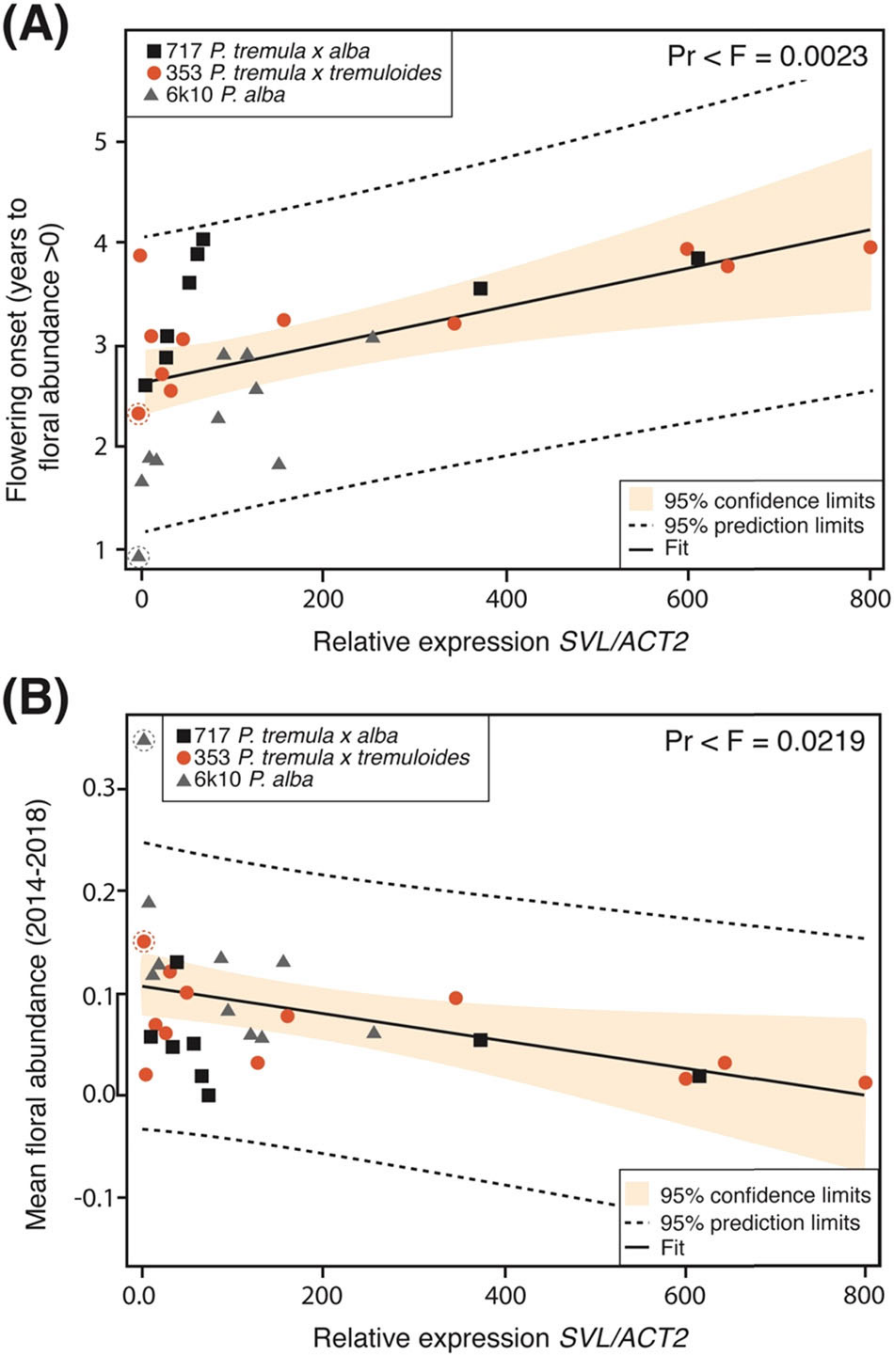
Regression analysis of flowering traits versus *SVL* expression in poplar transgenic and control lines (events). **A** Onset of flowering (in years) versus *SVL* gene expression relative to the *ACT2* housekeeping gene. **B** Floral abundance (floral abundance averaged over all years) versus *SVL* relative gene expression

Environmental variation within the transgenic field planting caused large variation in the rate of tree growth and thus onset of reproduction. To control for this, we performed a spatial adjustment on stem volume, as previously described^13^. To determine the usefulness of *SVL* for modifying reproduction, we used linear regression to test whether *SVL* expression was correlated with the onset or abundance of flowering. After initial analyses revealed that volume and floral traits were highly correlated, we used stem volume as a covariate in all regression analyses. We found a highly significant relationship between *SVL* expression and the onset of flowering (Pr < 0.0023) (Fig. 4A and Suppl. Table 2). We found a moderately significant relationship between *SVL* expression and floral abundance (catkin coverage in tree crown; see “Methods” section, Fig. 2) (Pr < 0.0219) (Fig. 4B).

**Fig. 5:**
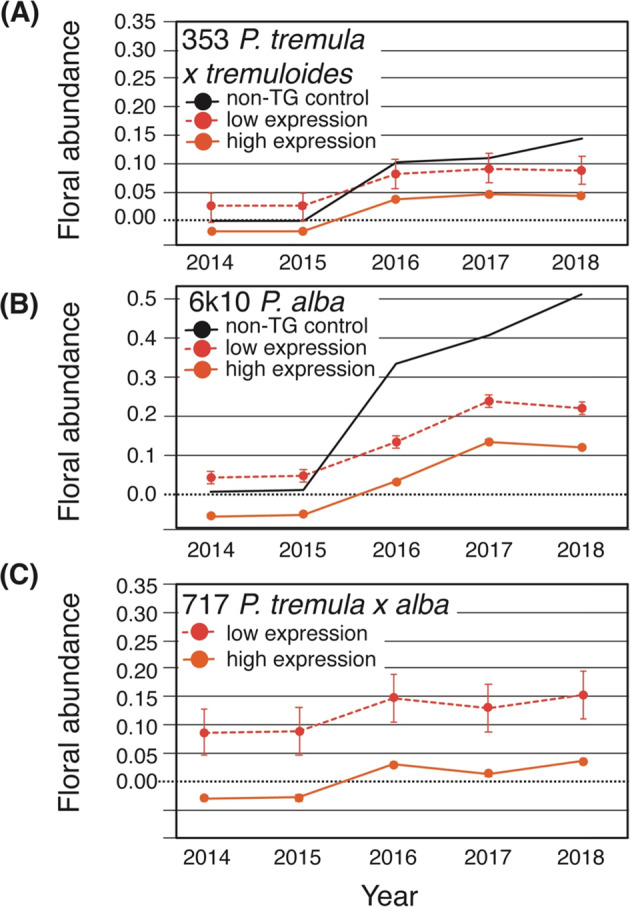
Floral abundance over time for low and high expression *35S:SVL* lines (events). Each point is the statistically adjusted (see methods) mean floral abundance of the three lowest or three highest *SVL* events per clone (353, 6k10, 717). Standard errors are represented by bracketed lines about the means. Some values are negative because they were predicted using a linear mixed effects model

Based on the molecular function of *SVL*, we predicted that high expression of *SVL* would delay flowering onset and abundance^33^. Thus, for each clone, we compared the floral abundance of the three highest and three lowest *SVL* expression events over time (Fig. 5). For each clone, the low-expression events had consistently greater estimated floral abundance (covariate-corrected for variation in volume index of each tree; Fig. 5). General inflorescence morphology of the *35S:SVL* transgenics was assessed in forced dormant cuttings in the 353 and 6k10 clones and was normal compared with controls (Suppl. Fig. 8A, B).

### *SVL* overexpressing trees had normal vegetative traits

Over the eight-year growth period, we collected data on stem volume, leaf area and mass, petiole length and width, and chlorophyll content. To investigate the effect of *SVL* expression on leaf vegetative characteristics, we analyzed the leaf phenotypes from 2018 using the same linear mixed effects model with stem volume as a covariate. Our regression analyses did not provide strong evidence for a statistically significant relationship between *SVL* expression and any of the leaf traits (Suppl. Table 1; all Pr ≥ 0.0975); examples of the statistical analysis employed is presented for ten traits in Suppl. Table 2. However, using the model without clones, the effects were significant for most traits at approximately the 10% level (Suppl. Table 1A). The vegetative traits varied widely between clones, but little within clones, as can be seen from the regression intercepts for petiole *L*:*W* ratio and leaf density in Suppl. Fig. 2A, B, respectively. Because volume was an important covariate in our statistical models, we used several approaches to investigate the potential effect of *SVL* expression on stem volume. First, we performed a regression analysis of stem volume versus *SVL* expression, ignoring clone (Suppl. Fig. 3A). Second, we performed the same analysis, but included clone as a fixed effect (Suppl. Fig. 3B). In both cases, there was not a statistically significant relationship (*P* > 0.13). For clone 717, there was weak tendency for a negative relationship between volume index and *SVL* expression; however, it clearly depended on a single event with very low volume and above average *SVL* expression (Suppl. Fig. 3B; Pr < 0.13).

### *35S:SVL* transgenics showed a weak and genotype-specific delay in floral and vegetative bud break

Though not a focus of our analysis, *SVL* has subsequently been reported to regulate vegetative bud break in *Populus*^28^. We undertook limited observations of bud break by scoring vegetative bud break over a 1-month period in 2014 and 2015 (Suppl. Fig. 4), by qualitatively scoring deviations from normal timing of floral bud break throughout the sterility trial (including other constructs) in 2018 (Suppl. Fig. 5), and by scoring detailed floral bud break morphology of 6K10 transgenics in the early spring of 2016 (Suppl. Fig. 6). In 717, but not in 353 or 6K10, we observed a delay in vegetative bud break in 2014 and 2015 in *35S:SVL* transgenics relative to controls (Suppl. Fig. 4). For floral bud break in 2018, 9% of all surviving trees in the sterility trial were scored as having abnormally late floral bud break. This included 9 *35S:SVL* transgenic ramets (5% of the 179 trees of this type in the trial), while the constructs with the strongest effects, PFPG and PLF (targeting *LEAFY* alone or in combination with *AGAMOUS* for knock-down using RNAi), had 6-fold higher rates of delayed flushing; 24% of their 210 and 188 trees, respectively, were scored as late (Suppl. Fig. 5). *35S:SVL* transgenics were 14th of 24 constructs in the sterility trial for late floral bud break, much below the prominently late PLF or PFPG constructs, and showed no association with *SVL* expression for those trees where expression was determined (Suppl. Fig. 5B, C). Based on detailed scoring of catkin bud morphology in 6K10 *35S:SVL* events and controls in 2016, we found a weak but statistically non-significant delay in floral bud break in transgenic events (Suppl. Fig. 6A); in addition, floral bud-break was poorly correlated with *SVL* expression in ramets where expression information was available, and generally less delayed than control trees (Suppl. Fig. 6B).

## Discussion

There have been many developments in the last two decades in molecular floral biology and genetic engineering technologies, and this study was undertaken throughout this period of rapid change. The *35S*:*SVL* construct was created and transformed into poplar in 2004 and 2005, prior to the publication of the first draft genome of *P. trichocarpa* and four years after the original characterization of *SVP* in Arabidopsis as a floral repressor^33,34^. The original transgenic trees were planted in an outdoor clone bank in the fall of 2007 and remained there until spring and summer of 2011, when a suitable large field trial site and long-term grant funding for the work became available. The trial trees were finally harvested in the spring and summer of 2019. Thus, this field trial represents nearly two decades of work toward testing the effectiveness of *SVP*-like genes for reproductive containment.

Because of the exploratory nature this work, there were a limited number of insertion events and trees per event. As a result, the statistical confidence in our results was also limited. For example, although the trend line amplitude (high vs. low predicted values) for the relationship between *SVL* expression and floral onset or abundance was 39% and 127% of the mean, respectively, the width of the confidence and the prediction brackets at the center of the regression graphs was a comparable 19 and 75% for onset, and 86 and 334% for abundance, respectively (Fig. 4). Thus, our ability to predict either flowering onset or abundance traits from gene expression was quite imprecise. However, when we pooled together the low vs. high expression events and examined their flowering behavior trends over several years of data, our prediction confidence improved. The predicted differences between the low and high expression groups of events were 114, 115, and 186% relative to the mean for the three clones (353, 6K10, 717, respectively), while the standard errors, averaged over the three clones, around the estimated floral abundance scores in each year, was a more modest 12% of the overall mean for the high expression event group and 86% for the low expression group.

We demonstrated that the overexpression of a floral transition repressor delays flowering in a long-lived forest tree (Fig. 4). Although these repressors have mostly been studied in annual plants, our work and recent studies in apple trees suggest they play comparable roles in mediating onset and abundance of reproduction in trees^19^. Our results are similar to those from a study of poplar RNAi transgenics, where suppression of *CEN1* was positively correlated with the onset and intensity of flowering over several years in the field^29^. However, *CEN1* overexpression transgenics showed delayed spring bud flush and greatly reduced growth in field conditions; thus, *CEN1* overexpression is not an option for genetic containment. Whereas *SVL* overexpression transgenics also showed delayed bud flush in controlled conditions, growth was not detectably reduced in our field trial^28^. Although photoperiod and temperature are major cues for phenology and manipulated to mimic the annual cycle, other environmental factors also influence phenology that perhaps activate genes and pathways that can bypass high levels of *SVL* to mediate dormancy release and bud flush in the field (reviewed in Brunner et al.)^35^. It is also possible that in a different climate, *35S:SVL* transgenics might show reduced growth. Together, these results illustrate the difficulty of predicting field performance from controlled environment studies. Delayed bud flush in an experimentally-induced annual cycle would suggest a negative growth effect in field conditions, but this was only the case for *CEN1* and not *SVL* overexpression.

To achieve substantial floral delay and repression, a very high and stable degree of overexpression appears to be needed; most transgenic events did not show a detectable reduction in flowering despite use of a very strong promoter. Only events with unusually high expression showed a useful delay in flowering and reduced flowering abundance (Fig. 3). Several events also showed large variation in *SVL* expression among ramets, which suggests suppression may have occurred in some branches (RNA was extracted from leaves of only one branch per tree). These findings are consistent with our observations of floral bud locations, which were sometimes located on a subset of very long branches rather than evenly distributed throughout the crown. Transgene silencing is commonly observed in transgenic plants that employ strong viral promoters and certain terminators, as were employed in our constructs (Fig. 1B)^36,37^. In other studies using such regulatory elements, however, stable gene overexpression or suppression has been observed over many years in most transgenic events in field-grown poplars^38^. Thus, it should be possible to pre-select events with strong and stable expression for commercial deployment. We also did not investigate the correlation between gene expression and SVL protein levels, which may also play a role in event phenotype, and whose expression could potentially be elevated through the use of improved 5′ UTR leaders such as the Arabidopsis Annexin and UP031279 5′ UTR leaders in the overexpression construct^39^. To save time and costs, initial pre-selection could be done in the laboratory and greenhouse, rather than in the field. However, given the propensity for transgene-mediated gene silencing, gene editing may offer greater potential than gene overexpression for successfully introducing stable changes in flowering phenotypes.

Gene editing may be accomplished by modifying regulatory regions of genes bound by transcription factors that inhibit *SVL* expression, or by inserting strong enhancers within *SVL* regulatory regions. Similarly, seasonal *FT1* production might be reduced by increasing the number of CArG-box *SVL* cis-elements in the promoter of *FT1*, thereby lowering flowering-inducing *FT1* production^28^. If temperature-dependent splicing is an important characteristic of *SVL*, as it is in Arabidopsis, it may be possible to increase repression by *SVL* by using gene editing to reduce non-functional splice variants^25^. These approaches may also cause fewer pleiotropic effects compared to constitutive overexpression.

Although not quantified in our vegetative trait analyses, we noticed some variation in crown structure that could not be measured due to the size of the trees. In addition, differences in several vegetative traits approached statistical significance (Suppl. Table 1B). Thus, further evaluation of *SVL* effects on crown structure and leaf/petiole morphology would be desirable. Such studies should also include block-plot yield studies (vs. single tree) of volume growth of non-flowering events, to see whether the delays in flowering can increase biomass productivity^7^. Although *SVL* and the similar *DAM* genes have been shown as having important roles in vegetative bud break from dormancy^26–28^, our limited analysis of vegetative and floral bud break suggests a weak and genotype specific interaction with *SVL* and this trait. We saw a clear difference in vegetative bud break in 717 transgenics vs. controls, but not in 353 or 6K10 (Suppl. Fig. 4). Additionally, compared to other constructs in our field study, *SVL* was not strongly represented when abnormally late vegetative bud flush was scored in 2018 (Suppl. Fig. 5).

**Fig. 6:**
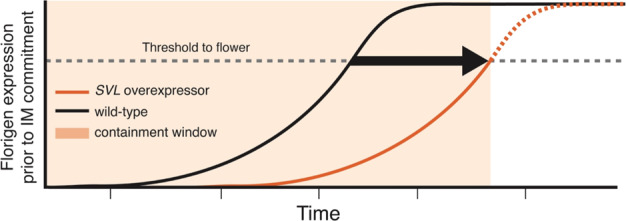
A delay in reproductive competency can provide a window of reproductive containment in forest trees. The acquisition of reproductive competency is different between wild-type trees and trees engineered with floral repression genes. A high degree of reproductive containment can be achieved as long as the trees are harvested before flowering occurs. The approximate delay in floral competency caused by increased *SVL* expression is represented by the black arrow, which spans between the wild-type (black solid line) and *SVL* overexpressor (orange hashed line) rates of florigen expression over time prior to reaching threshold levels for initiating reproductive development (gray hashed line). The approximate containment window afforded by *SVL* overexpression is indicated by the orange highlighted area. IM Inflorescence meristem

Although *SVL* overexpression had the intended effect of delaying flowering initiation and reducing floral abundance, it is unlikely to provide a comprehensive tool for reproductive containment, even in short rotation forest trees. First, in some short rotation species and genotypes, such as willows and coppice poplars, flowering occurs only 1–2 years after planting, thus *SVL* over-expression may have a shorter duration of benefit, perhaps insufficient to give both acceptable volume production and containment prior to harvest (Fig. 6). Second, even trees with high *SVL* expression eventually flowered to some degree. Given the potential for long distance gene dispersal from pollen and/or seed in poplars and some other tree species, even the dramatically reduced quantity of gene dispersal provided by *SVL* overexpression may be insufficient to meet regulatory or market demands. To provide high confidence in containment, we believe it would be best to combine *SVL* overexpression with other methods for transgene containment, particularly loss of function mutations in essential flowering genes enabled by gene editing methods^9^. With CRISPR, it is feasible to mutate multiple genes that affect flowering onset and fertility^40^.

We have shown that modification of *SVL* expression can be used to control flowering in poplar trees. However, deployment options will depend on continued innovations in gene editing and engineering technologies, the nature and location of tree production systems, and the laws and public views regarding gene dispersal from particular kinds of genetically modified trees.

## Materials and methods

### Construct assembly

The *SVL* gene from *Populus trichocarpa* (Potri.007G010800) was cloned into the overexpression vector pCAPO^8^. This vector contains a *35S* promoter to drive *SVL* expression, octopine synthase terminator, and *nptII* gene for kanamycin selection in plants. This vector backbone was previously described as pCAPT^31^.

### Poplar clones and genetic transformation

Three poplar clones were used to produce transgenic plants. A female hybrid clone, 717-1B4 *P. tremula x alba*, and a male hybrid clone, 353-38 *P. tremula x tremuloides*, were obtained from the Institute de la Recherche Agronomique (INRA) in Nancy, France. A second female clone, 6k10 *P. alba*, was originally obtained from the Università della Tuscia, in Viterbo, Italy^41^. Leaf disks and stem explants were transformed using *Agrobacterium*-mediated transformation as described previously^42^. Disarmed *Agrobacterium tumefaciens* strain AGL1 was used to generate all transgenic events.

### Field layout and experimental design

The *SVL*-overexpressing trees were part of a larger field planting, which contained many other constructs in three clonal backgrounds (each planted in separate blocks by clone). Tree transformation, propagation, site preparation, and field design have been described previously^8,13^. Individual transformation events were propagated to obtain multiple ramets per event, rooted, and transplanted to soil. Potted trees were placed in open-air outbuildings to acclimate, then planted in a clone bank at a field site near to Oregon State University (Corvallis) for nearly 4 years before collecting cuttings and rooted in preparation for planting in the field trial. Four ramets (genetically identical trees) were planted from each unique transformation event (hereafter referred to as an “event”) as a randomized split-plot design. The main-plots consisted of two replications of the three poplar clones (i.e., six clonal main-plots) established in a randomized design. The split-plots consisted of two-tree plots (specific gene insertion events, hereafter called events) established in a randomized design within each main-plot. Each split-plot consisted of one event (two adjacent ramets) representing one of 23 constructs in the larger field trial; the *35S:SVL* construct we studied is one of these 23 constructs^8^.

Because each event was derived from an individual transformation event, these events are considered biological replicates of the constructs, and for statistical analysis, event within clone and construct was considered a random effect. Main-plots also contained two-tree split-plots of control trees, which were non-transformed individuals of the same clonal background as the surrounding main-plot trees. The control trees were micropropagated and treated the same as the transgenic trees. Overall, we tested 11 transgenic events and one control event for poplar clone 353, 18 transgenic events and two control events for clone 6k10, and 16 transgenic events for clone 717.

The maximum number of live trees analyzed for growth and flowering traits per transgenic event was four. However, the control event for 717 had three ramets perish during the field trial, and the one ramet that survived had severely impaired growth. Thus, these four ramets were excluded from statistical analysis involving tree flowering or growth. Because we planted multiple split-plots of non-transgenic control trees, we analyzed growth and flowering using a total of nine control trees for clone 353, 51 control trees for clone 6k10, and no control trees for clone 717 (as discussed above).

Tree spacing was 2.3 m within rows and 2.3 m between rows, except for a 6.1 m spacing between every fourth row. Because trees were planted in a regularly-spaced grid, trees were identified by unique row and column numbers that facilitated spatial analysis, as discussed below. Weeds were controlled by installing shade cloth at the time of planting, and thereafter were controlled with a brush cutter and manual weeding. The trees were drip-irrigated immediately after planting in the summer of 2011, irrigated again the following summer, and then grown without irrigation in following years.

### Stem growth and leaf characteristics

Stem size was first measured in 2015. Tree height was measured using a height pole, and tree diameter was measured at knee height (diameter knee height, DKH) 45 cm above ground level, and at breast height (diameter breast height, DBH), 137 cm above ground level. Tree volume index (VOL), a measure of comparative stem size and mass, was calculated by summing the volume of a basal frustum and the volume of an upper cone. The volume of the frustum was calculated using the frustum height (92 cm, calculated from 137 cm − 45 cm), DKH, and DBH. The volume of the cone was calculated using the cone height (HT minus 137 cm) and DBH. Two leaves per tree were collected to measure total leaf chlorophyll leaf area, leaf mass, petiole length, and petiole width. At the time of leaf collection, trees were large enough to have their lower branches overlap. To avoid differences in leaf characteristics due to sun exposure, two leaves per tree were collected above the level of crown overlap on the south side of trees. Total leaf chlorophyll was measured three times per collected leaf using a hand-held SPAD meter (Konica Minolta). Collected leaves were also scanned with a flatbed scanner, and petiole lengths and widths were measured with digital calipers; leaves were then dried and weighed. Leaf area was measured using ImageJ software^43^. We used these measurements to evaluate the effects of *SVL* expression on stem volume, petiole length, petiole width, petiole length:width ratio, leaf area, leaf mass, and leaf density (leaf mass/leaf area).

### Floral measurements

Beginning in 2012, trees were observed each spring for the presence of floral buds and catkins, and then designated as flowering or non-flowering. From 2014 to 2018, this system was modified to include relative floral abundance (Fig. 2A)^13^. In brief, trees with no catkins or floral buds were scored 0, trees with very sparse catkins on a single branch were scored 1, trees with very sparse catkins on 2 or more branches were scored 2, trees with abundant catkins on less than 1/3 of potential crown locations were scored 3, trees with abundant catkins on 1/3 to 2/3 of potential crown locations were scored 4, and trees with abundant catkins on 2/3 or more of potential crown locations were scored 5. As explained below and shown in Fig. 2A, floral abundance score categories were converted into values that more closely represent the proportion of potential flowering locations that contained abundant catkins. Tree survival was also scored yearly in the spring based on vegetative bud flush. We used these measurements to evaluate the effects of *SVL* expression on floral abundance and floral onset.

### Gene expression analysis

Nine events each from the 717 and 6k10 backgrounds, and ten events from the 353 background were selected for gene expression analysis. Events were selected to be most different in initial flowering phenotypes, given that there were at least two ramets alive per event. Two ramets to represent each event from different blocks were subsequently selected for analysis. However, only one ramet was used for event #43 in the 717 background, due to degraded RNA in one of the ramet samples. Although this event is shown in Fig. 3, it was excluded from further statistical analysis.

During the spring of 2017, total RNA samples were extracted from healthy, fully expanded leaves that were collected from a single branch located on the south side of the tree from approximately 5 m above the ground. Leaves were held on ice for several hours, and then frozen at −80 °C until total RNA was extracted with a RNeasy kit (Qiagen). Following RNA quantification, cDNA was synthesized using a reverse transcription kit (Invitrogen). A qPCR analysis of *SVL* gene expression was conducted using an APbiosystems “Step One Plus” qPCR machine and SYBR green dye. Primers specific to *SVL* (5′-GGCAAGAGAGAGGATTCAGATAAA-3’, 5′- TGCTCCAGCTTCTCAAGATTC-3’) and an actin housekeeping gene (Potri. 004G153400, *ACT2*) (5′-CCCATTGAGCACGGTATTGT-3′, 5′-TACGACCACTGGCATACAGG-3′) were used to determine relative *SVL* gene expression. The *SVL* amplicon spanned exons 1 and 2 of the *P. trichocarpa SVL* native gene (the transgene construct had no intron). To determine if the native *SVL* transcript was amplified during our gene expression analysis, we determined the allele-specific sequences of the qPCR amplicons. For 717, this was determined from available haplo-specific genome sequences (http://aspendb.uga.edu/databases/spta-717-genome). For 6k10 and 353, genomic DNA was amplified using the primers (5′-ATAAGGCGCGCCTCCTGTCTTCTCACTCTTCCCATTG-3′, 5′-AATTGAGCTCGCACTGTCTTAAACACTCTCCACCA-3′), then digested and ligated between AscI and SacI restriction enzyme sites in a pUC19 derived plasmid. Individual clone insert sequences were determined by sanger sequencing until both alleles were found. The *SVL* qPCR primers perfectly matched both alleles in the *P. alba*, *P. tremula, and P. tremuloides* parental genomes in our transformed trees, thus the qPCR products for clone 353, 6k10, and 717 represented transgene and native *SVL* expression. The qPCR program consisted of an initial melt of 95 °C for 10 s, forty cycles of 95 °C for 15 s, and 60°C for 1 min, and a final melt curve analysis with a change of 0.3 °C s^−1^. Samples were run using three technical replicates, with sample *C*_T_ values computed as the average of the three technical replicates for *SVL* and *ACT2*. Relative *SVL* expression levels were determined using the 2^−ΔΔ*C*^_T_ method by comparing the *SVL* gene to the *ACT2* control^13,44^. To increase our capacity to detect a relationship between gene expression and flowering, events for gene expression analysis were selected to represent either the highest or lowest observed initial flowering onset within each clonal background. For qPCR, ten of eleven events (90%) were assayed for clone 353, while nine of eighteen events (50%) were assayed for clone 6k10, and nine of sixteen events (56%) were assayed for clone 717.

### Statistical analysis

Prior to data analysis, our categorical floral abundance measurements were converted into quantitative values that more closely reflected the proportion of potential flowering locations that contained abundant catkins (0 = 0, 1 = 0.025, 2 = 0.075, 3 = 0.215, 4 = 0.500, 5 = 0.835). Using the floral abundance data, we calculated floral onset, which is the year in which flowering was first recorded. Floral onset ranged from 0 for trees that were flowering when measurements were first made in 2014 to 5 for trees that flowered for the first time in 2019. For the 2015 volume index (VOL), we first performed a spatial adjustment on original data using the methods described by Klocko et al.^13,21^. This approach was used to mitigate the microenvironmental variation that affected growth rates of individual trees within the large clonal main-plots. We used the SAS GLIMMIX procedure to calculate residuals from a model that accounted for macroenvironmental variation among clonal main-plots (as a fixed effect), and microenvironmental variation by using the tree row and column positions. The final spatially-adjusted VOL data were calculated by adding the predicted grand mean to the individual-tree model residuals. We previously compared analyses of spatially adjusted versus unadjusted growth traits, and based on the Akaike Information Criterion (AIC) found that analyses of spatially adjusted data yielded a slightly better model fit (i.e., lower AIC)^13^.

Next, we analyzed all leaf and flowering traits using SAS PROC MIXED and the spatially adjusted VOL as a covariate. Because these data were unbalanced, we used the SAS MIXED procedure to calculate predicted values for all transgenic and control events using a model that included (1) the spatially adjusted VOL as a covariate, (2) clone, construct, and the clone × construct interaction as fixed effects, and (3) event within clone and construct and main-plot within clone as random effects. “Construct” was used to distinguish the transgenic versus non-transgenic control events. Using PROC MIXED, variance components for random effects were estimated using restricted maximum likelihood. The final gene expression data were completely balanced (i.e., two ramets/event), so we used event averages as the predicted expression value for each event.

We used SAS PROC REG to test for relationships between *SVL* gene expression versus stem volume, leaf traits, and flowering traits. The input data consisted of the event-level predicted values described above. The highly significant correlation between floral traits and stem volume prompted us to use volume as a covariate in these regression models. We used clone as a fixed effect in some of these regression models.

The details of each statistical model including covariates, fixed effects, random effects, and data outputs are given in Suppl. Table 2. For all analyses, assumptions of homogeneous variance and normality of errors were checked graphically with residual plots. All analyses were performed in SAS v9 (SAS Institute, Cary NC).

## Supplementary information

Supplemental figures
