# Supplementary material for: Overexpression of SHORT VEGETATIVE PHASE-LIKE (SVL) in Populus delays onset and reduces abundance of flowering in field-grown trees
Source: Hortic Res. 2021 Aug 1;8:167. doi: 10.1038/s41438-021-00600-4 (PMC8325693; doi:10.1038/s41438-021-00600-4)
Supplement: Supplementary file 1 — Supplemental figures [file 41438_2021_600_MOESM1_ESM.pdf]

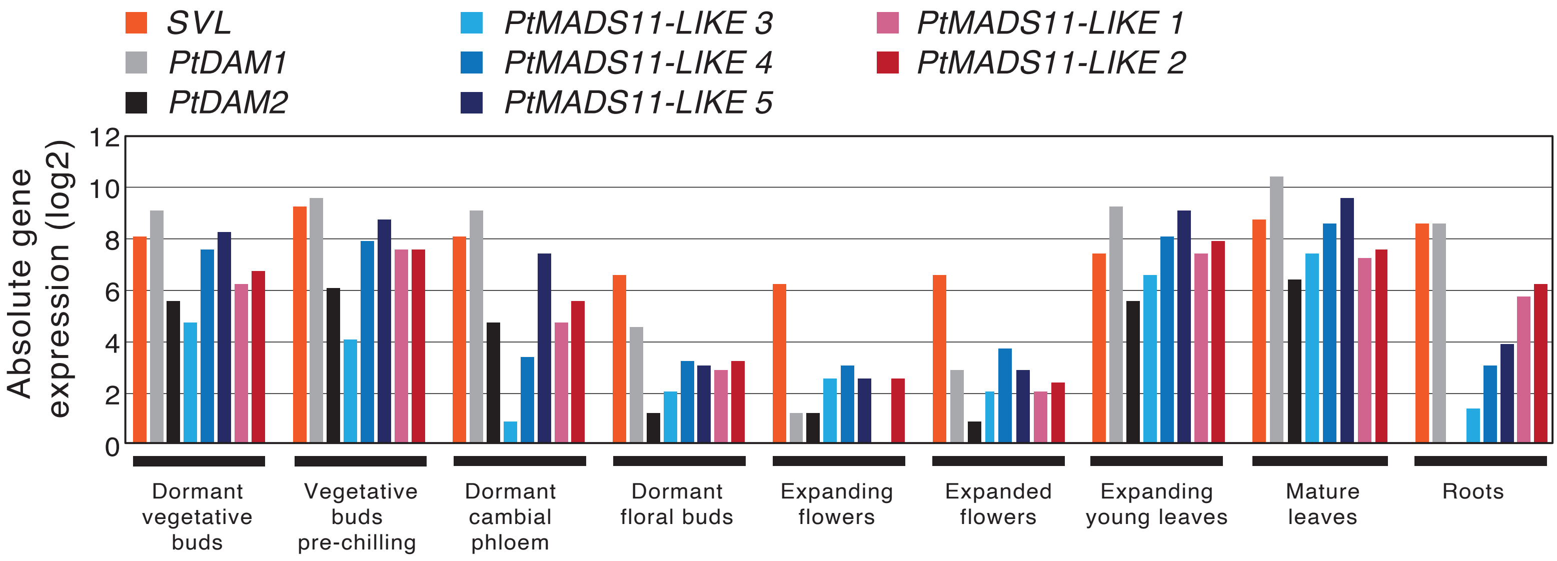

Supplemental Figure 1. Expression of *SVL* and closely related MADS-box genes in *Populus alba* samples compiled from publicly available RNA-seq datasets. Absolute gene expression (log2 transformed) for closely related MADS-box genes found in the *P. trichocarpa* reference genome (See Fig. 1). Values for several tissue types including (1) dormant vegetative buds (2) vegetative buds pre-chilling (3) dormant cambial phloem (4) dormant floral buds (5) expanding flowers (6) expanded flowers (7) expanding young leaves (8) mature leaves, and (9) roots, are shown. Histogram bars from left to right are in same order as genes listed at top of graph (from top to bottom in each column, in rows from left to right). All tissues were originally isolated from pooled samplings ($n > 3$) of tissues from a single *P. tremula* specimen in Umeå, Sweden between May and August 2010, with the exception of roots which were isolated from greenhouse grown clones of the same specimen during the same time period (Sundell *et. al,* 2015).

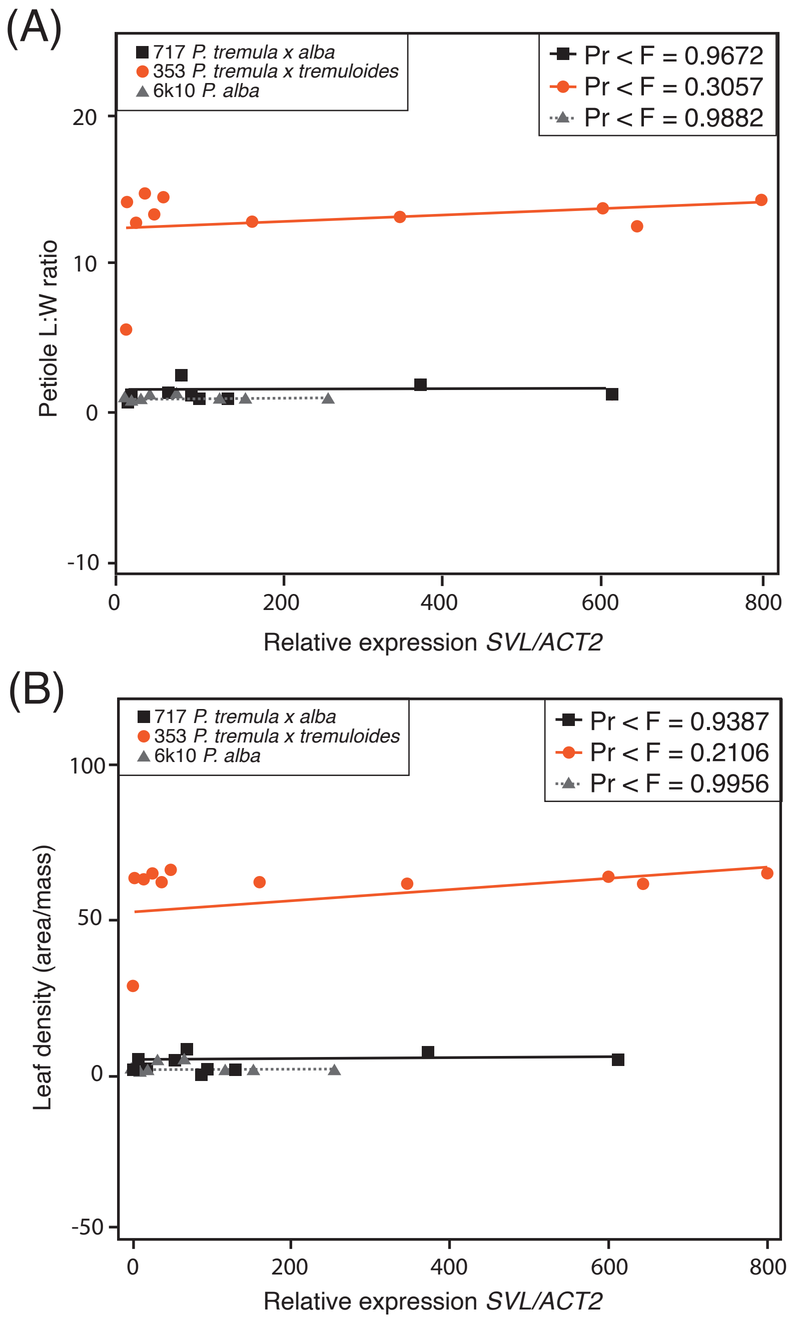

Supplemental Figure 2. Regression analysis of leaf traits versus *SVL* expression in transgenic and control lines (events). (A) Petiole length:width ratio versus *SVL* gene expression. **(B)** Leaf density versus *SVL* gene expression. Both analyses used clone (717, 353, 6k10) as a fixed effect and an independent slopes model. No significant relationship was found between leaf traits and *SVL* expression.

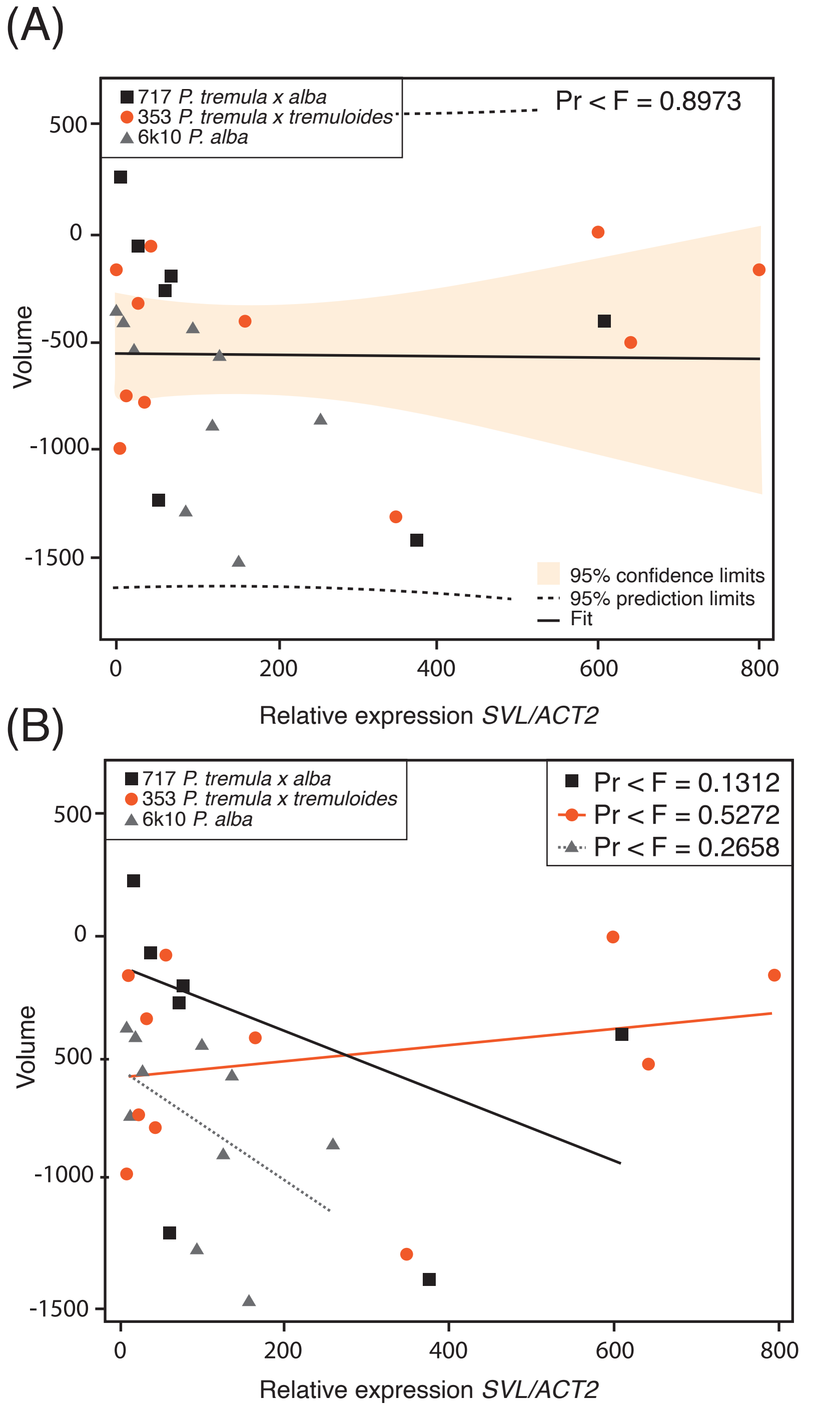

Supplemental Figure 3. Regression analysis of stem volume versus *SVL* expression in transgenic and control lines (events). (A) Stem volume versus *SVL* gene expression ignoring clone. **(B)** Stem volume versus *SVL* gene expression using clone as a fixed effect and an independent slopes model. No significant relationship was found between stem volume and *SVL* expression.

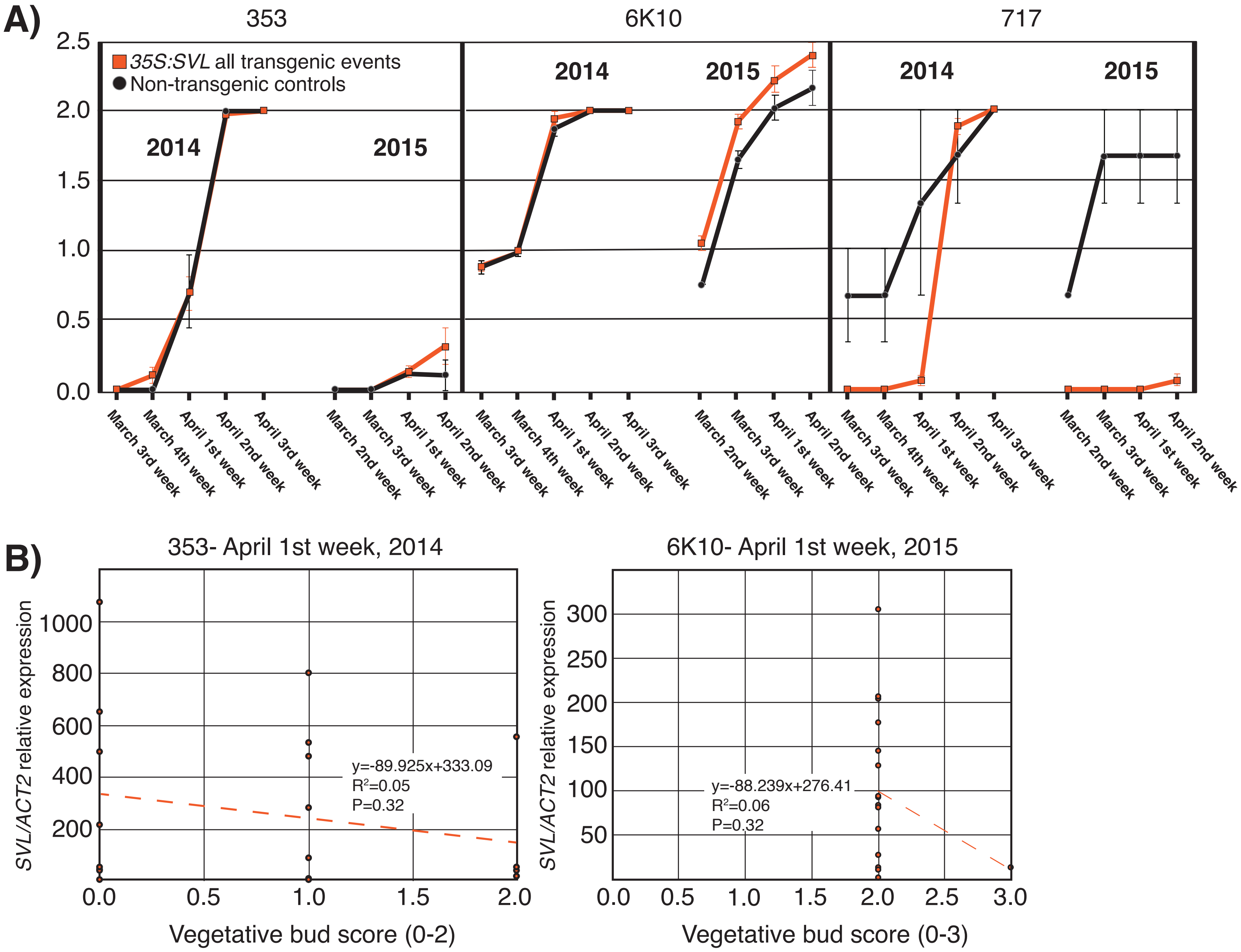

Figure S4: Vegetative bud break of *35S:SVL* transgenics and control trees in 2014 and 2015. A) Vegetative bud break was scored weekly beginning the third week of March, and the 2nd week of march in 2014 and 2015, respectively. A score of 0 equated to mainly unopened buds, a score of 1 equated to mostly elongating, swollen, but still unopened buds, and a score of 2 equated to mainly opened vegetative buds. In 2015 the scale was expanded to 3 to better encompass fully opened and expanded leaves. Points on each graph are averages of transgenic event means, with bars showing standard error. **B)** Vegetative bud break scores and *SVL* relative expression for each transgenic ramet where expression information was available. The 717 clone was excluded because of limited control expression and the experimental period did not capture 717 bud break adequately. Ramets without expression data are not plotted. Regression trendlines are plotted on each axis, with the R^2 and accompanying p-values.

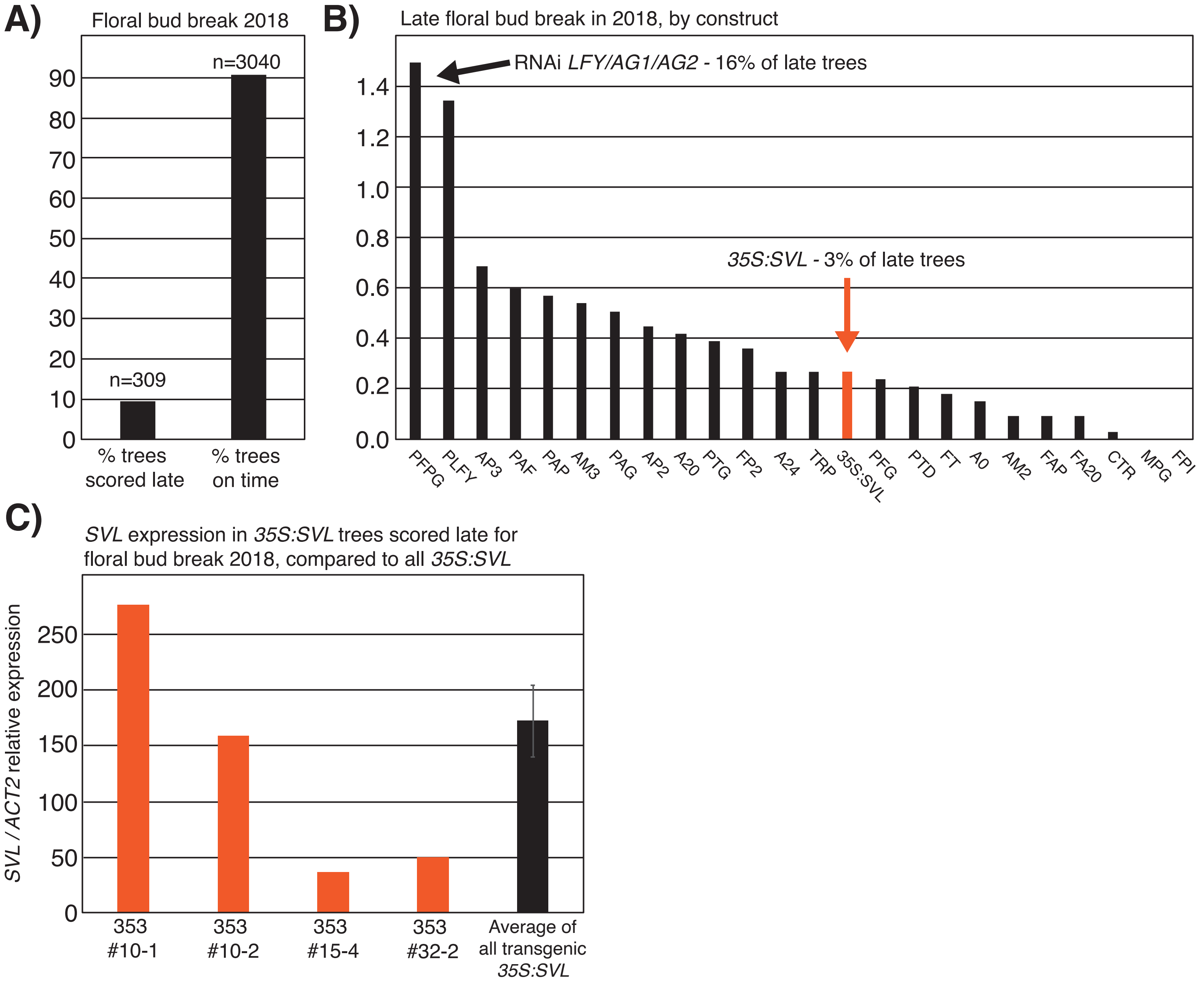

Figure S5: Late floral bud break across all constructs in the sterility trial in 2018 . A) Floral bud break was scored in early spring 2018, and individual trees were noted as early, not scored, or late. Histogram plot of all trees in the plantation scored as having late floral bud break. **B)** Percentage of trees overall by construct which were scored late for floral bud break in 2018. Abbreviations are as follows: PFPG = RNAi LFY/AG1/AG2, PLFY = RNAi LFY, AP3 = dominant negative AtAP1-M3, PAF = RNAi LFY, AP1-1,AP1-2, PAP = RNAi AP1-1/AP1-2, AM3 = dominant negative AtAG, PAG = RNAi AG1/AG2/AP1-1/AP1-2, AP2 - dominant negative AtAP1-M2, A20 = RNAi AGL20, PTG = RNAi AG1/AG2, FP2 = RNAi PFPFL2, A24 = RNAi AGL24, TRP = RNAi LFY/AG1/AG2/AP1-1/AP1-2, PS = 35S:SVL, RNAi LFY/AG1/AG2, PTD = RNAi AP3, FT = RNAi FT1/FT2, A0 = 35S:AGL24, AM2 = dominant negative AtAG, AM2 = dominant negative AtAG, FAP = RNAi FT1/FT2/AGL20/FPFL1, FA20 = RNAi FT1/FT2/AGL20, CTR = control, MPG = RNAi AG1/AG2 eith Rb7 MAR, FPI = RNAi PFPFL1. **C)** *SVL* relative expression of individual trees which were scored as having late floral bud break in 2018, compared to the overall mean of all transgenic trees sampled for *SVL* expression in all clones. 5 other trees were scored as late for floral bud break, which belonged to 3 transgenic events (6k10 #20-3, 717 #116-3 and #116-4, and 717 #163-3, and #163-4, for which no *SVL* expression data is available.

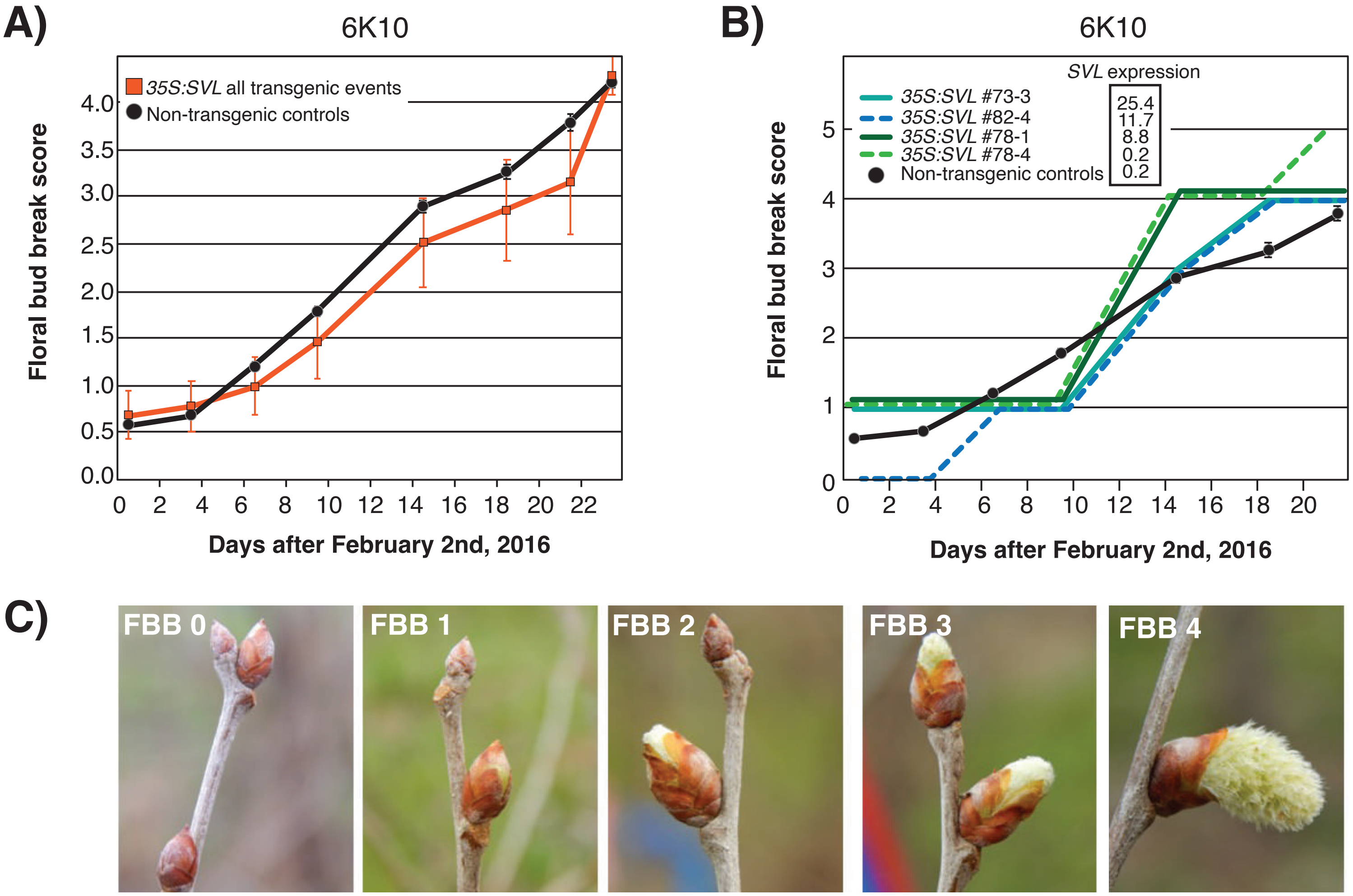

Figure S6: Floral bud break of *35S:SVL* transgenics and control trees 6K10 clone trees in 2016. A) Floral bud break was scored every several days beginning on February 2nd 2016. *35S:SVL* floral bud scores are averages of all event means, regardless of whether expression information was known. Standard error is shown computed among all ramets in controls and on event means for the *35S:SVL* transgenics. **B)** Floral bud break scores for each transgenic ramet scored where expression information was available, shown about the control bud break scores. *SVL* relative expression levels for each ramet and averaged among the control 6k10 samples tested are shown in the upper legend. **C)** Floral bud break scoring key in 6k10 control trees with relevant developmental stages for the period displayed shown.

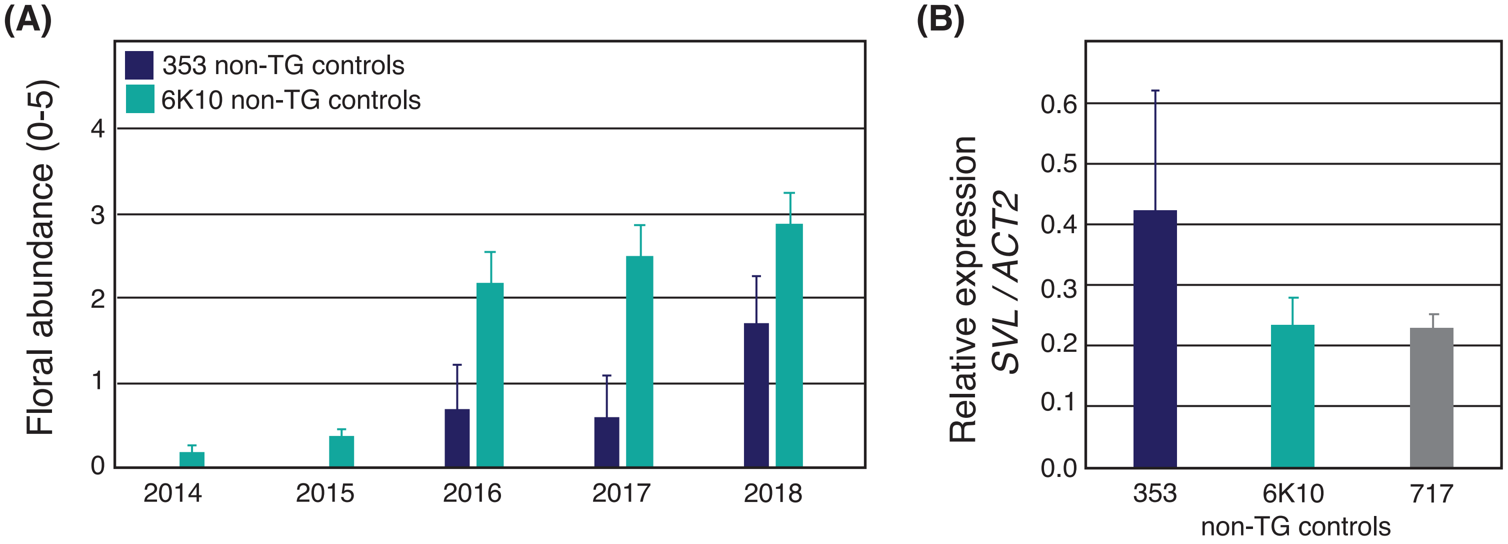

Figure S7. Floral abundance and *SVL* expression in non-transgenic control trees. A) mean floral abundance of non-transgenic controls (non-TG control), by year recorded from 2014-2018 among trees surviving the trial. Data shown are means of 10 353 and 31 6k10 control trees, and error bars show standard error. Only a single 717 control tree survived the trial and never flowered. Examples of floral score ratings are shown in Figure 2. **B)** Relative expression of *SVL* in non-transgenic control leaf tissue. Bars are means of two biological replicates, except in 717 where two RNA samples were exctracted from one individual surviving tree (All data inputs are means of technical triplicates). Error bars depict standard error. A comparison of expression in controls to *35S:SVL* events is shown in Figure 3.

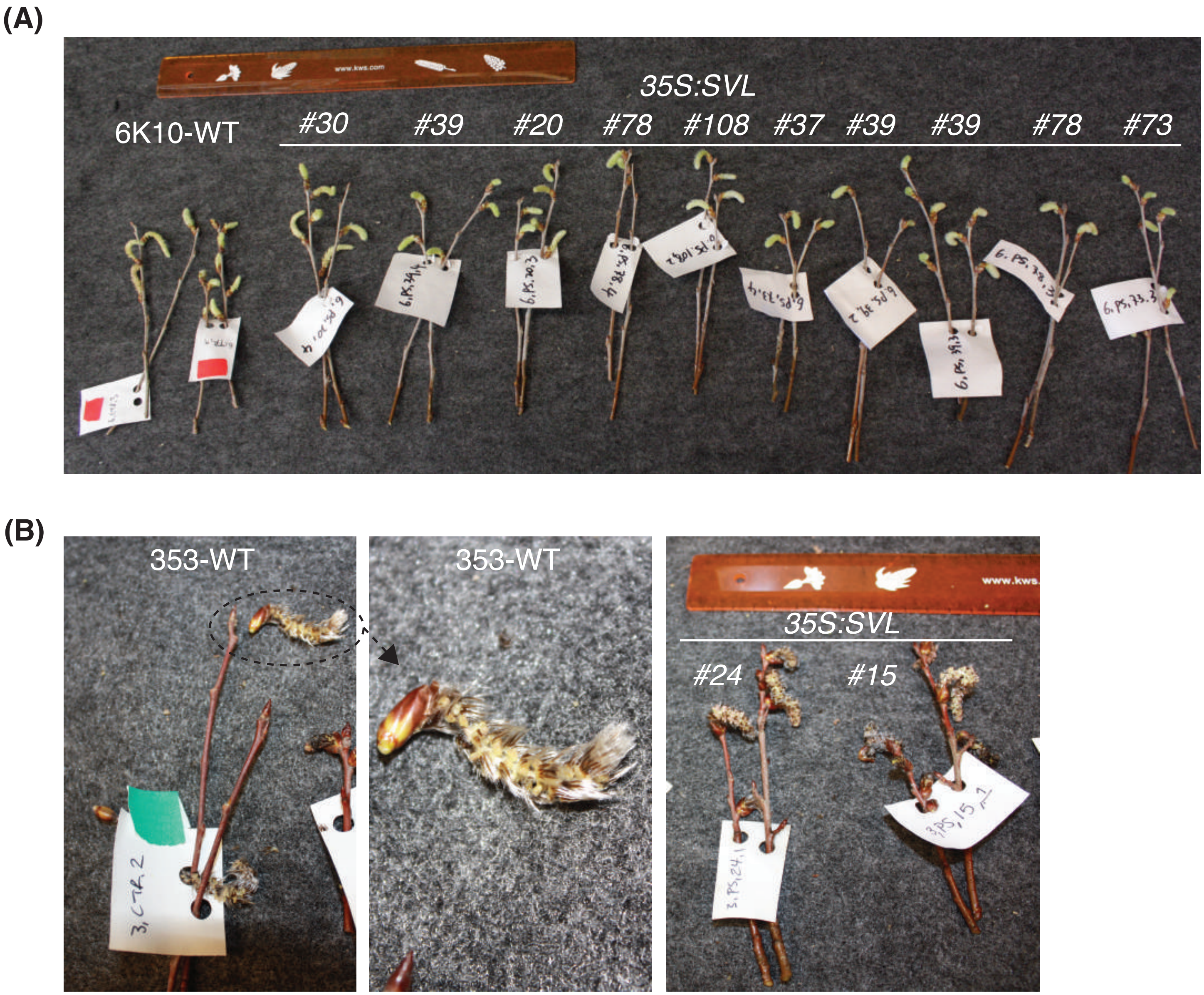

Figure S8: Floral morphology of *35S:SVL* transgenics and 6k10 / 353 control trees in 2015 and 2016. **A)** Young female catkins of *35S:SVL* transgenics and control 6k10 forced cuttings in early 2015. **B)** Mature male catkins of *35S:SVL* transgenics and control 353 forced cuttings in early 2016. In both clones floral morphology was normal compared with controls in all trangenic events. 717 floral morphology not assayed in the *35S:SVL* transgenic events.

A) Not including clone in model

Analysis (single trait vs. *SVL* expression)	Pr < F
Petiole length	0.0980
Petiole width	0.0975
Leaf area	0.0981
Leaf mass	0.0975
SPAD	0.0979
Volume	0.8973

B) Including clone in model

	Pr < F		
Analysis (ratio trait or volume vs. *SVL* expression)	Clone 353	Clone 6k10	Clone 717
Petiole length : width ratio	0.3057	0.9882	0.9672
Leaf density	0.2106	0.9956	0.9387
Volume (no covariate)	0.5272	0.2658	0.1323

Supplementary Table 1. Summary of P-values for vegetative trait analyses against *SVL* expression using mixed model analyses in SAS. (A) P-values for regression analyses of single vegetative traits compared with *SVL* expression, which did not include clonal genetic background. **(B)** P-values for regression analyses of ratio vegetative traits or volume compared with *SVL* expression, which included clonal genetic background. P-values are listed for clone 353, 6k10, and 717.

Supplementary Table 2. Results of analyses of flowering and growth traits compared against *SVL* expression level. Results from mixed model analyses in SAS. Fixed effects, random effects, and covariates are listed at the beginning of each analysis. AIC is the Akaike Information Criterion used to judge model fit, covariance parameter estimates are the variances estimated for random effects, the Type III tests show the F-value and associated probability for differences among the treatment means, and least square means for treatments are shown with their standard errors. Regression analysis with ANOVA show the F-value and associated probability for differences among the treatment means for *SVL* expression against other traits. Floral traits such as onset and abundance use multiple years of floral data, spatially adjusted volume data are computed from 2015 DBH and height measurements, and leaf traits/chlorophyll contents are all from 2018 acquired data. Abbreviations are Cov Parm, covariance parameters; Flower tmt, flower treatment; Num DF, numerator degrees of freedom; and Den DF, denominator degrees of freedom.

Flowering onset

Covariates: Spatially-adjusted volume
Fixed Effects: Spatially adjusted volume, clone (353, 6k10, 717), construct (control, 35S:SVL), clone x construct
Random Effects: Transgenic event, block

**AIC**	825.3

Covariance Parameter Estimates

Covariance Parameter	Estimate
Transgenic event	0.4583
Block	2.4789
Residual	1.3454

Type III Tests of Fixed Effects

Effect	Num DF	Den DF	F value	Pr > F
Spatially-adjusted volume	1	182	35.92	0.0001
Clone	2	5	0.47	0.6492
Construct	1	44	3.37	0.0733
Clone x construct	1	182	0.81	0.3682

Least Squares Means

Effect	Construct	Clone	Estimate	Standard Error
**Clone**		353	3.9334	1.1792
**Clone**		6k10	2.7592	0.8105
**Clone**		717	no est.	N/A
**Construct**	CTR		no est.	N/A
**Construct**	35S:SVP		4.1503	0.6143
**Clone x construct**	CTR	353	3.433	1.3351
**Clone x construct**	35S:SVP	353	4.4338	1.141
**Clone x construct**	CTR	6k10	2.0111	0.8737
**Clone x construct**	35S:SVP	6k10	3.5073	0.8861
**Clone x construct**	35S:SVP	717	4.5097	1.1354

Regression Analysis
Model= Model 1
Dependent variable = P-onset of flowering

ANOVA

Source	DF	Sum of Squares	Mean Square	F value	Pr > F
**Model**	1	5.37426	5.37426	11.35	0.0023
**Error**	27	12.78285	0.47344		
**Corrected total**	28	18.15711			

**Observations**	29
**Parameters**	2
**Error DF**	27
**MSE**	0.4734
**R-square**	0.296
**Adjusted R-square**	0.2699

Flowering intensity

Covariates: Spatially-adjusted volume
Fixed Effects: Spatially adjusted volume, clone (353, 6k10, 717), construct (control, 35S:SVL), clone x construct
Random Effects: Transgenic event, block

**AIC**	-32.7

Covariance Parameter Estimates

Covariance Parameter	Estimate
Transgenic event	0.004835
Block	0.03733
Residual	0.03534

Type III Tests of Fixed Effects				
Effect	Num DF	Den DF	F value	Pr > F
Spatially-adjusted volume	1	174	50.75	0.0001
Clone	2	5	0.29	0.7613
Construct	1	44	4.17	0.0471
Clone x construct	1	174	3.8	0.0529

Least Squares Means				
Effect	Construct	Clone	Estimate	Standard Error
**Clone**		353	0.09309	0.1455
**Clone**		6k10	0.21172	0.0988
**Clone**		717	no est.	N/A
**Construct**	CTR		no est.	N/A
**Construct**	35S:SVP		0.06528	0.0754
**Clone x construct**	CTR	353	0.13	0.166
**Clone x construct**	35S:SVP	353	0.05614	0.1408
**Clone x construct**	CTR	6k10	0.3406	0.1048
**Clone x construct**	35S:SVP	6k10	0.0937	0.1077
**Clone x construct**	35S:SVP	717	0.04601	0.1399

Regression Analysis
Model= Model 1
Dependent variable = P-flowering intensity

ANOVA					
Source	DF	Sum of Squares	Mean Square	F value	Pr > F
**Model**	1	0.02576	0.02576	5.92	0.0219
**Error**	27	0.11752	0.00435		
**Corrected total**	28	0.14329			

**Observations**	29
**Parameters**	2
**Error DF**	27
**MSE**	0.0044
**R-square**	0.1798
**Adjusted R-square**	0.1494

Petiole length

Petiole Length 2018
Covariates: Spatially-adjusted volume
Fixed Effects: Spatially adjusted volume, clone (353, 6k10, 717), construct (control, 35S:SVL), clone x construct
Random Effects: Transgenic event, block

**AIC**	3228.3

Covariance Parameter Estimates	
Covariance Parameter	Estimate
Transgenic event	2051.16
Block	0
Residual	55373

Type III Tests of Fixed Effects				
Effect	Num DF	Den DF	F value	Pr > F
Spatially-adjusted volume	1	182	0.14	0.7061
Clone	2	5	29.99	0.0016
Construct	1	44	3.52	0.0674
Clone x construct	1	182	4.92	0.0271

Least Squares Means				
Effect	Construct	Clone	Estimate	Standard Error
**Clone**		353	672.77	47.722
**Clone**		6k10	27.4307	27.4307
**Clone**		717	no est.	N/A
**Construct**	CTR		no est.	N/A
**Construct**	35S:SVP		19.6004	19.6004
**Clone x construct**	CTR	353	87.9418	87.9418
**Clone x construct**	35S:SVP	353	37.5253	37.5253
**Clone x construct**	CTR	6k10	46.5447	46.5447

Clone x construct	35S:SVP	6k10	29.4134	29.4134
Clone x construct	35S:SVP	717	32.8028	32.8028

Regression Analysis
Model= Model 1
Dependent variable = P-petiole length 2018

ANOVA					
Source	DF	Sum of Squares	Mean Square	F value	Pr > F
Model	1	121204	121204	2.94	0.098
Error	27	1114021	41260		
Corrected total	28	1235225			

Observations	29
Parameters	2
Error DF	27
MSE	41260
R-square	0.0981
Adjusted R-square	0.0647

Petiole width

Petiole Width 2018
Covariates: Spatially-adjusted volume
Fixed Effects: Spatially adjusted volume, clone (353, 6k10, 717), construct (control, 35S:SVL), clone x construct
Random Effects: Transgenic event, block

AIC	3252

Covariance Parameter Estimates	
Covariance Parameter	Estimate
Transgenic event	2284.32
Block	0
Residual	61331

Type III Tests of Fixed Effects				
Effect	Num DF	Den DF	F value	Pr > F
Spatially-adjusted volume	1	182	0.14	0.7069
Clone	2	5	29.99	0.0016
Construct	1	44	3.58	0.0649
Clone x construct	1	182	5.07	0.0255

Least Squares Means				
Effect	Construct	Clone	Estimate	Standard Error
Clone		353	656.11	50.246
Clone		6k10	995.71	28.8984
Clone		717	no est.	N/A
Construct	CTR		no est.	N/A
Construct	35S:SVP		832.88	20.6357
Clone x construct	CTR	353	775.37	92.5954
Clone x construct	35S:SVP	353	536.85	39.505
Clone x construct	CTR	6k10	996.11	49.0484
Clone x construct	35S:SVP	6k10	995.31	30.9654
Clone x construct	35S:SVP	717	966.47	43.5337

Regression Analysis
Model= Model 1
Dependent variable = P-petiole width 2018

ANOVA					
Source	DF	Sum of Squares	Mean Square	F value	Pr > F
Model	1	135120	135120	2.95	0.0975
Error	27	1238136	455857		
Corrected total	28	1373256			

Observations	29
Parameters	2
Error DF	27
MSE	45857
R-square	0.0984
Adjusted R-square	0.065

Petiole length : width ratio

Petiole length : width ratio 2018

Covariates: Spatially-adjusted volume

Fixed Effects: Spatially adjusted volume, clone (353, 6k10, 717), construct (control, 35S:SVL), clone x construct

Random Effects: Transgenic event, block

**AIC**	1585

Covariance Parameter Estimates	
**Covariance Parameter**	**Estimate**
Transgenic event	2.1081
Block	0
Residual	44.7514

Type III Tests of Fixed Effects

Effect	Num DF	Den DF	F value	Pr > F
Spatially-adjusted volume	1	182	0.41	0.5225
Clone	2	5	27.65	0.002
Construct	1	44	4.38	0.0422
Clone x construct	1	182	6.9	0.0094

Least Squares Means

Effect	Construct	Clone	Estimate	Standard Error
**Clone**		353	10	1.3855
**Clone**		6k10	1.1106	0.8185
**Clone**		717	no est.	N/A
**Construct**	CTR		no est.	N/A
**Construct**	35S:SVP		5.2875	0.5675
**Clone x construct**	CTR	353	5.8108	2.5563
**Clone x construct**	35S:SVP	353	13.3032	1.0834
**Clone x construct**	CTR	6k10	1.1332	1.4059
**Clone x construct**	35S:SVP	6k10	1.088	0.8495
**Clone x construct**	35S:SVP	717	1.4712	0.9476

Regression Analysis

Model= Independent slopes (SAS transreg proc)

Dependent variable = P-petiole length : width ratio 2018

Univariate ANOVA

Source	DF	Sum of Squares	Mean Square	F value	Pr > F
**Model**	5	941.542	188.3084	68.67	0.0001
**Error**	23	63.073	2.7423		
**Corrected total**	28	1004.615			

**Observations**	29
**Parameters**	6
**Error DF**	23
**Root MSE**	1.65599
**R-square**	0.9372
**Adjusted R-square**	0.9236
**Dependent mean**	5.77197
**Coefficient of variation**	28.69029

Univariate regression table based on usual degrees of freedom

Source	DF	Coefficient	Sum of Squares (type II)	Mean Square	F value	Pr > F
**clone (717)**	1	1.6065636	13.1	13.1	4.78	0.0393
**clone (6k10)**	1	1.0812491	5.18	5.18	1.89	0.1824
**clone (353)**	1	12.6034213	1021.64	1021.64	372.55	0.0001
**clone (717) x SVL expression**	1	0.0001192	0	0	0	0.9672
**clone (6k10) x SVL expression**	1	0.000101	0	0	0	0.9882
**clone (353) x SVL expression**	1	0.0018159	3.01	3.01	1.1	0.3057

Leaf mass

Leaf mass 2018

Covariates: Spatially-adjusted volume

Fixed Effects: Spatially adjusted volume, clone (353, 6k10, 717), construct (control, 35S:SVL), clone x construct

Random Effects: Transgenic event, block

**AIC**	3242.5

Covariance Parameter Estimates	
**Covariance Parameter**	**Estimate**
Transgenic event	2197.56

Block	0
Residual	58861

Type III Tests of Fixed Effects

Effect	Num DF	Den DF	F value	Pr > F
Spatially-adjusted volume	1	182	0.16	0.6913
Clone	2	5	30.24	0.0016
Construct	1	44	3.58	0.065
Clone x construct	1	182	5.09	0.0253

Least Squares Means

Effect	Construct	Clone	Estimate	Standard Error
**Clone**		353	661.94	49.2334
**Clone**		6k10	995.7	28.3232
**Clone**		717	no est.	N/A
**Construct**	CTR		no est.	N/A
**Construct**	35S:SVP		836.08	20.2193
**Clone x construct**	CTR	353	778.86	90.7306
**Clone x construct**	35S:SVP	353	545.03	38.7069
**Clone x construct**	CTR	6k10	996.01	48.0779
**Clone x construct**	35S:SVP	6k10	995.39	30.3399
**Clone x construct**	35S:SVP	717	967.82	33.8362

Regression Analysis

Model= Model 1

Dependent variable = P-leaf mass 2018

ANOVA

Source	DF	Sum of Squares	Mean Square	F value	Pr > F
**Model**	1	135651	135651	2.95	0.0975
**Error**	27	1242877	46032		
**Corrected total**	28	1378528			

**Observations**	29
**Parameters**	2
**Error DF**	27
**MSE**	46032
**R-square**	0.0984
**Adjusted R-square**	0.065

Leaf area

Leaf area 2018

Covariates: Spatially-adjusted volume

Fixed Effects: Spatially adjusted volume, clone (353, 6k10, 717), construct (control, 35S:SVL), clone x construct

Random Effects: Transgenic event, block

**AIC**	3252

Covariance Parameter Estimates

Covariance Parameter	Estimate
Transgenic event	2284.32
Block	0
Residual	61331

Type III Tests of Fixed Effects

Effect	Num DF	Den DF	F value	Pr > F
Spatially-adjusted volume	1	182	0.14	0.7069
Clone	2	5	29.99	0.0016
Construct	1	44	3.58	0.0649
Clone x construct	1	182	5.07	0.0255

Least Squares Means

Effect	Construct	Clone	Estimate	Standard Error
**Clone**		353	656.11	50.246
**Clone**		6k10	995.71	28.8984
**Clone**		717	no est.	N/A
**Construct**	CTR		no est.	N/A
**Construct**	35S:SVP		832.88	20.6357
**Clone x construct**	CTR	353	775.37	92.5954
**Clone x construct**	35S:SVP	353	536.85	39.505
**Clone x construct**	CTR	6k10	996.11	49.0484
**Clone x construct**	35S:SVP	6k10	995.31	30.9654
**Clone x construct**	35S:SVP	717	966.47	43.5337

Regression Analysis

Model= Model 1

Dependent variable = P-leaf area 2018

ANOVA					
**Source**	**DF**	**Sum of Squares**	**Mean Square**	**F value**	**Pr > F**
**Model**	1	130178	130178	2.94	0.0981
**Error**	27	1197063	44336		
**Corrected total**	28	1327241			

**Observations**	29
**Parameters**	2
**Error DF**	27
**MSE**	44336
**R-square**	0.0981
**Adjusted R-square**	0.0647

Leaf density

Leaf density 2018

Covariates: Spatially-adjusted volume

Fixed Effects: Spatially adjusted volume, clone (353, 6k10, 717), construct (control, 35S:SVL), clone x construct

Random Effects: Transgenic event, block

**AIC**	2325.5

Covariance Parameter Estimates	
**Covariance Parameter**	**Estimate**
Transgenic event	24.0579
Block	0
Residual	1123.73

Type III Tests of Fixed Effects				
**Effect**	**Num DF**	**Den DF**	**F value**	**Pr > F**
Spatially-adjusted volume	1	182	0.23	0.6357
Clone	2	5	29.33	0.0017
Construct	1	44	4.38	0.0422
Clone x construct	1	182	5.69	0.0181

Least Squares Means				
**Effect**	**Construct**	**Clone**	**Estimate**	**Standard Error**
**Clone**		353	46	6.5508
**Clone**		6k10	1.3651	3.5852
**Clone**		717	no est.	N/A
**Construct**	CTR		no est.	N/A
**Construct**	35S:SVP		22.8354	2.7091
**Clone x construct**	CTR	353	28.9402	12.0394
**Clone x construct**	35S:SVP	353	62.3241	5.2112
**Clone x construct**	CTR	6k10	1.4918	5.9299
**Clone x construct**	35S:SVP	6k10	1.2384	4.0827
**Clone x construct**	35S:SVP	717	4.9437	4.552

Regression Analysis

Model= Independent slopes (SAS transreg proc)

Dependent variable = P-leaf density 2018

Univariate ANOVA					
**Source**	**DF**	**Sum of Squares**	**Mean Square**	**F value**	**Pr > F**
**Model**	5	22602.53	4520.507	98	0.0001
**Error**	23	1060.91	46.127		
**Corrected total**	28	23663.45			

**Observations**	29
**Parameters**	6
**Error DF**	23
**Root MSE**	6.79166
**R-square**	0.9552
**Adjusted R-square**	0.9454
**Dependent mean**	24.82938
**Coefficient of variation**	27.35334

Univariate regression table based on usual degrees of freedom

Source	DF	Coefficient	Sum of Squares (type II)	Mean Square	F value	Pr > F
clone (717)	1	5.2711659	141	141	3.06	0.937
clone (6k10)	1	1.2272801	6.7	6.7	0.14	0.07071
clone (353)	1	58.1963082	21782.7	21782.7	472.24	0.0001
clone (717) x SVL expression	1	0.0009143	0.3	0.3	0.01	0.9387
clone (6k10) x SVL expression	1	0.0001531	0	0	0	0.9956
clone (353) x SVL expression	1	0.0091566	76.5	76.5	1.66	0.2106

SPAD

Covariates: Spatially-adjusted volume
Fixed Effects: Spatially adjusted volume, clone (353, 6k10, 717), construct (control, 35S:SVL), clone x construct
Random Effects: Transgenic event, block

AIC	3233.1

Covariance Parameter Estimates	
**Covariance Parameter**	**Estimate**
Transgenic event	2165.09
Block	0
Residual	56468

Type III Tests of Fixed Effects

Effect	Num DF	Den DF	F value	Pr > F
Spatially-adjusted volume	1	182	0.14	0.7096
Clone	2	5	29.93	0.0017
Construct	1	44	3.58	0.0651
Clone x construct	1	182	5.09	0.0252

Least Squares Means

Effect	Construct	Clone	Estimate	Standard Error
Clone		353	670.07	48.3277
Clone		6k10	995.79	27.882
Clone		717	no est.	N/A
Construct	CTR		no est.	N/A
Construct	35S:SVP		839.44	19.8411
Clone x construct	CTR	353	784.9	89.0734
Clone x construct	35S:SVP	353	555.24	37.9717
Clone x construct	CTR	6k10	996.25	47.392
Clone x construct	35S:SVP	6k10	995.34	29.7647
Clone x construct	35S:SVP	717	967.75	33.1952

Regression Analysis
Model= Model 1
Dependent variable = P-SPAD 2018

ANOVA

Source	DF	Sum of Squares	Mean Square	F value	Pr > F
Model	1	124516	124516	2.94	0.0979
Error	27	1143766	42362		
Corrected total	28	1268282			

Observations	29
Parameters	2
Error DF	27
MSE	42362
R-square	0.0982
Adjusted R-square	0.0648

Volume

Volume
Covariates: No covariate
Fixed Effects: Clone (353, 6k10, 717), construct (control, 35S:SVL), clone x construct
Random Effects: Transgenic event, block

AIC	5210.5

Covariance Parameter Estimates	
**Covariance Parameter**	**Estimate**
Transgenic event	906678
Block	1518677
Residual	4490854

Type III Tests of Fixed Effects				
Effect	Num DF	Den DF	F value	Pr > F
Clone	2	9	0.13	0.8785
Construct	1	44	1.22	0.2751
Clone x construct	1	230	0.41	0.5232

Least Squares Means				
Effect	Construct	Clone	Estimate	Standard Error
Clone		353	-1,097	908.46
Clone		6k10	-1519.59	676.65
Clone		717	no est.	N/A
Construct	CTR		no est.	N/A
Construct	35S:SVP		-722.58	449.87
Clone x construct	CTR	353	-1737.6	1270.47
Clone x construct	35S:SVP	353	-456.66	810.16
Clone x construct	CTR	6k10	-1864.28	895.88
Clone x construct	35S:SVP	6k10	-1174.9	708.4
Clone x construct	35S:SVP	717	-536.19	789.65

Volume Analysis 1

Covariates: Spatially-adjusted volume
Fixed Effects: Spatially adjusted volume, construct (control, 35S:SVL)
Random Effects: Transgenic event, block

Regression Analysis

Model= Model 1
Dependent variable = P-spatially adusted volume

ANOVA					
Source	DF	Sum of Squares	Mean Square	F value	Pr > F
Model	1	4660.1421	4660.1421	0.02	0.8973
Error	27	7414930	274627		
Corrected total	28	7419590			

Observations	29
Parameters	2
Error DF	27
MSE	274627
R-square	0.0006
Adjusted R-square	-0.036

Volume Analysis 2

Covariates: none
Fixed Effects: Spatially adjusted volume, clone (353, 6k10, 717), construct (control, 35S:SVL), clone x construct
Random Effects: Transgenic event, block

Regression Analysis

Model= Independent slopes (SAS transreg proc)
Dependent variable = P-spatially adjusted volume

Univariate ANOVA					
Source	DF	Sum of Squares	Mean Square	F value	Pr > F
Model	5	1872330	374465.9	1.55	0.213
Error	23	5547260	241185.2		
Corrected total	28	7419590			

Observations	29
Parameters	6
Error DF	23
Root MSE	491.10613
R-square	0.2523
Adjusted R-square	0.0898
Dependent mean	-547.02386
Coefficient of variation	-89.77783

Univariate regression table based on usual degrees of freedom						
Source	DF	Coefficient	Sum of Squares (type II)	Mean Square	F value	Pr > F
clone (717)	1	-130.23701	86103	86103	0.36	0.556
clone (6k10)	1	-569.9611	1440421	1440421	5.97	0.0226
clone (353)	1	-579.46375	2159605	2159605	8.95	0.0065
clone (717) x SVL expression	1	-1.3269	587181	587181	2.43	0.1323
clone (6k10) x SVL expression	1	-2.2854	313766	313766	1.3	0.2658
clone (353) x SVL expression	1	0.33007	99417	99417	0.41	0.5272
